# Intravenous Immunoglobulin Alone for Coronary Artery Lesion Treatment of Kawasaki Disease: A Randomized Clinical Trial

**DOI:** 10.1001/jamanetworkopen.2025.3063

**Published:** 2025-04-03

**Authors:** Ho-Chang Kuo, Ming-Chih Lin, Chung-Chih Kao, Ken-Pen Weng, Yun Ding, Zhi Han, Chih-Jung Chen, Sheng-Ling Jan, Kuang-Jen Chien, Chun-Hsiang Ko, Chien-Yu Lin, Wei-Te Lei, Mindy Ming-Huey Guo, Kuender D. Yang, Karl G. Sylvester, John C. Whitin, Lu Tian, Henry Chubb, Scott R. Ceresnak, Doff McElhinney, Harvey J. Cohen, Xuefeng B. Ling

**Affiliations:** 1 College of Medicine, Chang Gung University, Taoyuan, Taiwan; 2 Department of Pediatrics and Kawasaki Disease Center, Kaohsiung Chang Gung Memorial Hospital, Kaohsiung, Taiwan; 3 Department of Respiratory Therapy, Kaohsiung Chang Gung Memorial Hospital, Kaohsiung, Taiwan; 4 Chang Gung University College of Medicine, Kaohsiung, Taiwan; 5 Department of Post-Baccalaureate Medicine, College of Medicine, National Chung Hsing University, Taichung, Taiwan; 6 Children’s Medical Center, Taichung Veterans General Hospital, Taichung, Taiwan; 7 School of Medicine, National Yang Ming Chiao Tung University, Taipei, Taiwan; 8 Department of Food and Nutrition, Providence University, Taichung, Taiwan; 9 School of Medicine, Chung Shan Medical University, Taichung, Taiwan; 10 Department of Pediatric Cardiology, Tungs’ Taichung Metro Harbor Hospital, Taichung, Taiwan; 11 Congenital Structural Heart Disease Center, Department of Pediatrics, Kaohsiung Veterans General Hospital, Kaohsiung, Taiwan; 12 School of Medicine, Stanford University, Stanford, California; 13 Division of Pediatric Infectious Diseases, Department of Pediatrics, Linkou Chang Gung Memorial Hospital, Taoyuan, Taiwan; 14 Molecular Infectious Diseases Research Center, Chang Gung Memorial Hospital, Taoyuan, Taiwan; 15 Department of Pediatrics, Children’s Medical Center, Taichung Veterans General Hospital, Taichung, Taiwan; 16 Department of Pediatrics, School of Medicine, National Yang Ming Chiao Tung University, Taipei, Taiwan; 17 Department of Pediatrics, School of Medicine, Kaohsiung Medical University, Kaohsiung, Taiwan; 18 Section of Immunology Rheumatology and Allergy, Department of Pediatrics, Hsinchu Mackay Memorial Hospital, Hsinchu, Taiwan; 19 MacKay Children’s Hospital, Taipei, Taiwan

## Abstract

**Importance:**

Aspirin (acetylsalicylic acid) and intravenous immunoglobulin (IVIG) are standard treatments for Kawasaki disease (KD) to reduce coronary artery lesions (CALs). However, the optimal duration and dosage of aspirin remain inconsistent across hospitals. The absence of large-scale, multicenter randomized clinical trials hinders a clear understanding of the effectiveness of high-dose aspirin.

**Objective:**

To evaluate the effectiveness of IVIG alone compared with IVIG combined with high-dose aspirin as the active interventional therapy for KD and to compare treatment effectiveness across various KD subgroups.

**Design, Setting, and Participants:**

In this prospective, evaluator-blinded, multicenter noninferiority randomized clinical trial, children (aged <6 years) who had been diagnosed with KD according to American Heart Association criteria were recruited from 5 medical centers in Taiwan and were enrolled between September 1, 2016, and August 31, 2018, with follow-up assessments at 6 weeks and 6 months after treatment. Data were analyzed between January 23, 2023, and January 29, 2024.

**Intervention:**

The standard group received IVIG (2 g/kg) plus high-dose aspirin (80-100 mg/kg per day) until fever subsided for 48 hours. The intervention group received IVIG (2 g/kg) alone.

**Main Outcomes and Measures:**

The primary outcome was the occurrence of CALs at 6 weeks. The noninferiority margin was set at 10%. Data analysis was performed using χ^2^ tests for categorical variables; independent *t* tests for continuous, normally distributed variables; generalized estimating equations for variables without specific distributions at multiple time points; and repeated-measures analysis of variance for continuous variables at multiple time points.

**Results:**

The final cohort consisted of 134 patients with KD (mean [SD] age, 1.8 [1.3] years; 82 males [61.2%]), with matched age, weight, height, and sex distributions in 2 groups. Overall, in the IVIG plus aspirin group, among 69 patients, CAL occurrence decreased from 9 (13.0%) at baseline to 2 (2.9%) at 6 weeks and to 1 (1.4%) at 6 months. In the IVIG-only group, among 65 patients, CAL occurrence decreased from 7 (10.8%) at diagnosis to 1 (1.5%) at 6 weeks and to 2 (3.1%) at 6 months. No statistically significant differences in CAL frequency were observed between the 2 groups (0.7 percentage points [95% CI, −4.5 to 5.8 percentage points]; *P* = .65). There were also no significant differences in the treatment or prophylactic effect.

**Conclusions and Relevance:**

This randomized clinical trial demonstrated the noninferiority of IVIG alone compared with IVIG plus aspirin, with a noninferiority margin set at 10%. The findings suggest that addition of high-dose aspirin during initial IVIG treatment is not clinically meaningful for CAL reduction in children with KD. Future studies on IVIG treatment alone for CAL reduction in KD across diverse racial and ethnic groups, beyond the Asian population, may be necessary to confirm minimal racial and ethnic variability and the broad applicability of these findings.

**Trial Registration:**

ClinicalTrials.gov Identifier: NCT02951234

## Key Points

**Question:**

Does the combination of high-dose aspirin and intravenous immunoglobulin (IVIG) provide greater benefit than IVIG alone in preventing the development of coronary artery lesions (CALs) among children with Kawasaki disease?

**Findings:**

In this noninferiority randomized clinical trial of 134 children with Kawasaki disease, the rate of CAL reduction in the IVIG plus aspirin group decreased from a baseline of 13% to 3% at 6 weeks; in the IVIG-only group, the rate of CAL reduction decreased from a baseline of 11% to 2% at 6 weeks. No statistically significant difference was observed between the 2 groups.

**Meaning:**

The findings of this study suggest that addition of high-dose aspirin to IVIG treatment is not clinically meaningful for the reduction of CALs among children with Kawasaki disease.

## Teaser

This randomized clinical trial assessed whether a combination of high-dose aspirin and intravenous immunoglobulin provides greater benefit than intravenous immunoglobulin alone in preventing the development of coronary artery lesions in Kawasaki disease.

## Introduction

Kawasaki disease (KD) is an acute vasculitis characterized by self-limiting tendencies, predominantly impacting medium-sized vessels and resulting in coronary artery aneurysms, the most concerning complication of coronary artery lesions (CALs),^1,2^ which includes dilatations, fistulas, infarctions,^3^ and aneurysms.^4^ Initial treatment typically includes intravenous immunoglobulin (IVIG) and high-dose aspirin (acetylsalicylic acid; 80-100 mg/kg per day), which synergistically exert anti-inflammatory effects according to the American Heart Association (AHA) criteria. It is noteworthy that high-dose aspirin, with its anti-inflammatory properties, primarily addresses inflammation, while lower doses are primarily directed at thrombosis.

Studies suggest that administering IVIG at a dose of 2 g/kg per day is highly successful in minimizing the likelihood of CALs in individuals with KD.^5,6,7,8^ The approved therapy for the acute phase of KD, as recommended by the AHA and the American Academy of Pediatrics, includes the administration of IVIG with a single 2-g/kg dose given over 10 to 12 hours along with an oral moderate dose of aspirin at 30 to 50 mg/kg per day and a high dose at 80 to 100 mg/kg per day.^9^ Although aspirin demonstrates notable anti-inflammatory effects at high doses and antiplatelet effects at low doses, to our knowledge, there is no prospective study validating its role in diminishing the formation of CALs. Several studies propose a strong correlation between the incidence of CALs and the dosage of IVIG, indicating that the dosage of aspirin may not have a significant influence.^5,10,11^ In a retrospective analysis by investigators of our team, which included 851 patients with KD from 2 medical centers in Taiwan, no noteworthy distinctions between groups (with or without high-dose aspirin) with regard to sex, IVIG resistance, CAL formation, and the duration of hospitalization were found.^8^

At present, there is a shortage of well-conducted randomized clinical trials that can determine whether high-dose aspirin should continue to be a fundamental element of the treatment protocol for children diagnosed with KD.^12,13,14^ According to Sanati et al,^13^ in a prospective open-label trial conducted at a single site with a small-sized cohort, administering the standard 2 g/kg per day IVIG without high-dose aspirin during acute-stage therapy did not elevate the risk of CAL formation in 62 patients with both typical and atypical KD.

The risk associated with aspirin therapy in children with KD is generally considered low and appears to be comparable to risks reported in other situations. However, documented cases exist of severe adverse effects associated with aspirin in children undergoing KD treatment.^8,15,16,17^ Given the potential risks of drug toxicity and the limited robust evidence for aspirin in the prevention of CAL formation, there is a necessity to reevaluate the role of high-dose aspirin during the acute stage of KD. To address this concern, we conducted a multicenter, prospective, parallel-group, open-label randomized clinical trial to investigate whether high-dose aspirin, administered during the acute stage of KD, shows noninferiority compared with IVIG alone in preventing CAL formation.

A recent investigative study, exploring the heterogeneity of KD, identified 4 unique patient clusters. These clusters were delineated according to subjective clinical features and laboratory results, resulting in the formation of the liver subgroup, the band subgroup, the node subgroup, and the young subgroup.^18^ It is noteworthy that these subgroups exhibited differences in their response to treatment and the outcomes of the disease, including the risk of coronary artery aneurysm and the rate of IVIG resistance. Therefore, in this trial, we implemented an approach considering these subgroups following a recent proposal by Wang et al.^18^ The goal was to compare the treatment effectiveness for each subgroup of KD between the standard and test groups.

## Methods

This randomized clinical trial was approved by the institutional review board at each site of the participating institutions, including Linkou Chang Gung Memorial Hospital, Kaohsiung Chang Gung Memorial Hospital, Taichung Veterans General Hospital, Kaohsiung Veterans General Hospital, and Tungs’ Taichung Metro Harbor Hospital. Written informed consent was obtained from a legal guardian. The study followed the Consolidated Standards of Reporting Trials (CONSORT) reporting guideline.

### Overall Study Design

**Figure 1.**
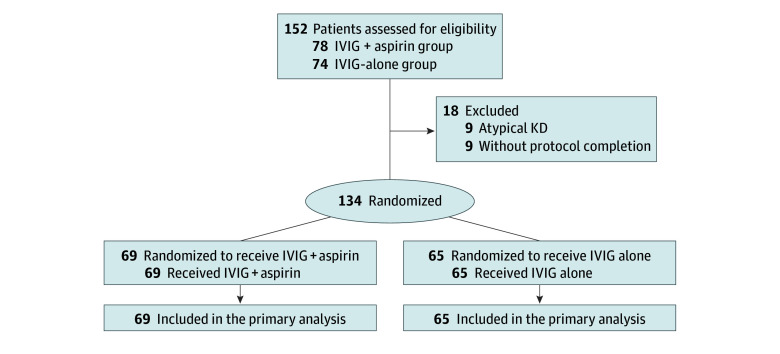
Flow Diagram for Patient Enrollment IVIG indicates intravenous immunoglobulin; KD, Kawasaki disease.

This study was structured as a multicenter, prospective, evaluator-blinded, noninferiority randomized clinical trial with 2 parallel groups. The trial protocol is provided in Supplement 1, and additional detailed information is provided in the eMethods in Supplement 2. The aim was to evaluate the effectiveness of IVIG alone in comparison with IVIG combined with high-dose aspirin (80-100 mg/kg per day) as the primary treatment for KD during the acute stage. Children (aged <6 years) who had been diagnosed with KD according to AHA criteria were eligible and were recruited from 5 medical centers in Taiwan. Patients were enrolled between September 1, 2016, and August 31, 2018, with follow-up assessments at 6 weeks and at 6 months after enrollment (the enrollment flowchart is provided in Figure 1).

**Figure 2.**
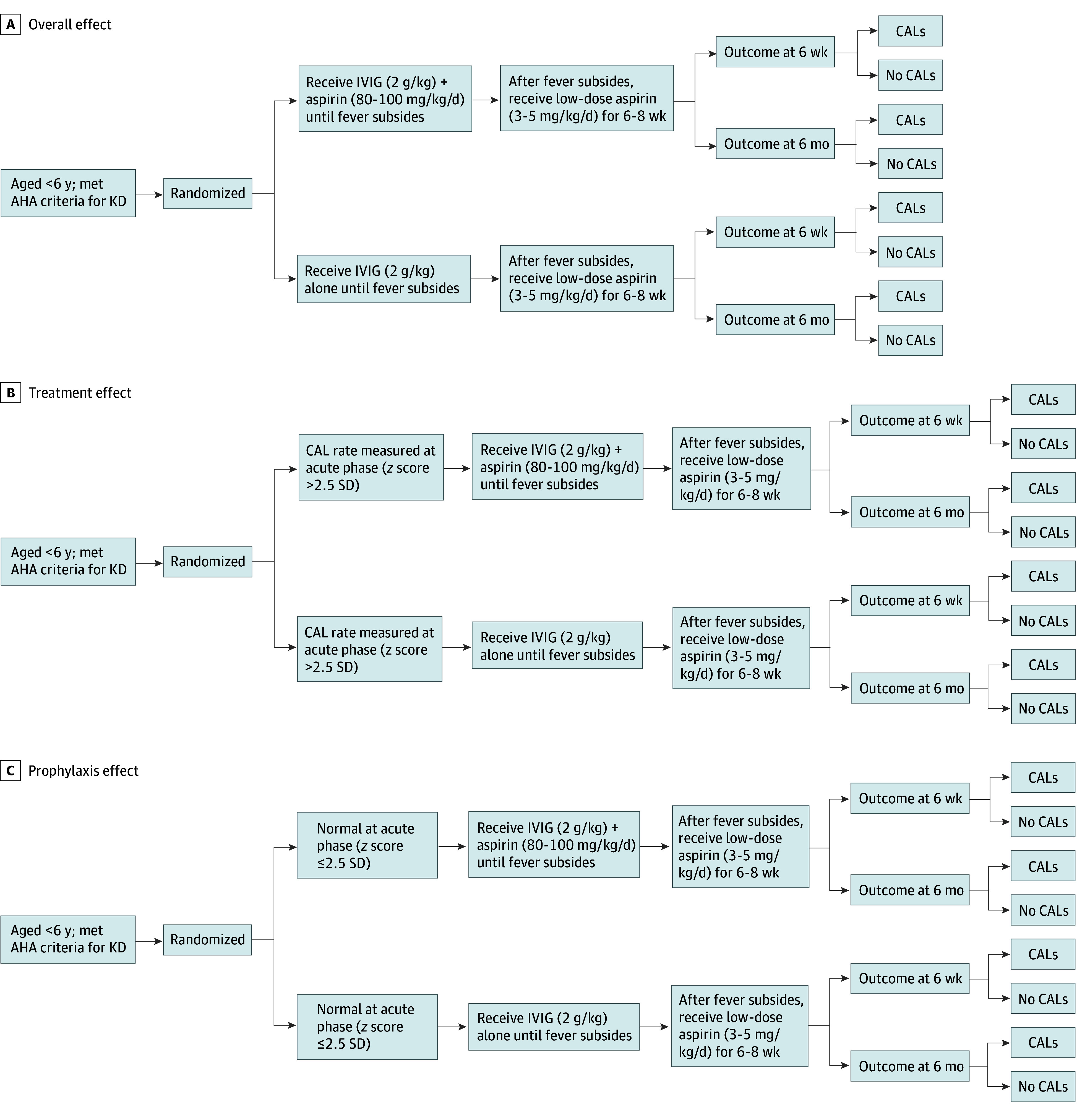
Schematic Experiment Design AHA indicates American Heart Association; CAL, coronary artery lesion; IVIG, intravenous immunoglobulin; KD, Kawasaki disease.

Individuals with a fever lasting more than 10 days or a history of prior treatment with steroids or biologics were excluded. The experimental flow of the trial is shown in Figure 2. All patients were administered a dose of IVIG at 2 g/kg over a 12-hour duration, either with high-dose aspirin (80-100 mg/kg per day) in the IVIG plus aspirin group or without aspirin in the IVIG-alone group. Following the resolution of fever, low-dose aspirin (3-5 mg/kg per day) was prescribed for all patients with KD for a period of 6 weeks or until a normal echocardiogram and normal inflammation laboratory data were achieved, in accordance with the guidelines provided by the AHA.

### Outcome Assessments and Evaluation

The primary end point was CAL formation at 6 weeks after enrollment. CAL formation was defined as a luminal diameter exceeding 3.0 mm in children younger than 5 years or exceeding 4.0 mm in those aged 5 years or older, when the internal diameter of a segment was 1.5 times or greater than that of an adjacent segment, or when the luminal contour was distinctly irregular, with a *z* score of more than 2.5 SDs. The body weight and height used for calculating the *z* score were obtained from the Taiwan Society of Pediatric Cardiology.^19^

### Statistical Analysis

Data were analyzed between January 23, 2023, and January 29, 2024. All baseline characteristics were used to assess the comparability of both arms of the trial. The primary outcome evaluation and safety assessment were conducted using the intention-to-treat population. Continuous variables, including age, sex, hospitalization duration (days), fever duration (days), height, weight, and clinical manifestations, are presented as either means (SDs) or frequency tables. Data analysis was performed using χ^2^ tests for categorical variables; independent *t* tests for continuous, normally distributed variables; generalized estimating equations for variables without specific distributions at multiple time points; and repeated-measures analysis of variance for continuous variables at multiple time points. A 2-sided *P* value ≤ .05 was considered statistically significant. A 97.5% CI for the difference in heart lesion between the IVIG-alone group and the IVIG plus aspirin group was constructed. If the upper end of the 97.5% CI was below 10%—the prespecified, noninferiority margin—then the noninferiority of the overall effect, combining both treatment and prophylactic effects, on CAL formation was established. Statistical analyses were performed using SAS, version 9.4 (SAS Institute Inc) and Prism software, version 10.1.0 (GraphPad).

## Results

### Coronary Artery Changes and Frequency

In total, 152 patients diagnosed with KD were enrolled. Of these, 78 patients were assigned to the IVIG plus aspirin (standard) group, and 74 were assigned to the IVIG-alone (intervention) group. In the IVIG plus aspirin group, 4 patients with atypical KD and 5 patients who did not complete the study protocol were excluded, whereas in the IVIG-alone group, 5 patients with atypical KD and 4 patients without protocol completion were excluded. After excluding a total of 18 patients, 134 patients with KD (mean [SD] age, 1.8 [1.3] years; 52 females [38.8%] and 82 males [61.2%]) were enrolled in the final cohort and randomly assigned to the IVIG plus aspirin group (n = 69) and the IVIG-alone group (n = 65) for further analysis. Baseline characteristics are presented in eAppendix 1 and eTable 1 in Supplement 2. Patients were matched by age, weight, height, and sex distributions in the 2 groups.

As presented in Table 1, 9 of 69 patients (13.0%) in the IVIG plus aspirin group and 7 of 65 patients (10.8%) in the IVIG-alone group presented an abnormal coronary artery diameter (*z* score >2.5 SD) in at least 1 of the 3 coronary arteries. Among the 69 patients in the IVIG plus aspirin group, these percentages decreased to 2.9% (n = 2 patients) at 6 weeks and 1.4% (n = 1 patient) at 6 months. Among the 65 patients in the IVIG-alone group, these percentages decreased to 1.5% (n = 1 patient) at 6 weeks and 3.1% (n = 2 patients) at 6 months. No significant differences were observed between the groups regarding the frequency of coronary artery abnormalities during the study period (0.7 percentage points [95% CI, −4.5 to 5.8 percentage points]; *P* = .65). The frequency of CALs exhibited a significant decrease compared with the baseline, starting from week 6 through the end of the study period at month 6. Similarly, no significant differences were observed between the 2 groups on either the main left coronary artery (LCA) or the right coronary artery (RCA) (detailed findings are described in eAppendix 2 in Supplement 2).

**Table 1.** Frequency of Abnormal Coronary Artery Diameter by CAL Type and Time Point: Overall Effect^a^

CAL type	Time point								Overall *P* value^c^
	Baseline		At 6 wk			At 6 mo			
	IVIG alone (n = 65)	IVIG plus aspirin (n = 69)	IVIG alone (n = 65)	IVIG plus aspirin (n = 69)	*P* value^b^	IVIG alone (n = 65)	IVIG plus aspirin (n = 69)	*P* value^b^	
LCA	4 (6.2)	6 (8.7)	0	2 (2.9)	.02	1 (1.5)	0	.006	.45
LAD	0	0	0	0	NA	0	0	NA	NA
RCA	4 (6.2)	3 (4.3)	1 (1.5)	1 (1.4)	.09	1 (1.5)	1 (1.4)	.09	.36
Overall	7 (10.8)	9 (13.0)	1 (1.5)	2 (2.9)	.002	2 (3.1)	1 (1.4)	.002	.65

Abbreviations: CAL, coronary artery lesion; IVIG, intravenous immunoglobulin; LAD, left anterior descending; LCA, main left coronary artery; NA, not applicable; RCA, right coronary artery.

^a^ Data are presented as No. (%) of patients.

^b^ CAL frequency difference compared with baseline.

^c^ CAL frequency changes between IVIG-alone and IVIG plus aspirin groups for the entire study period.

As for the noninferiority comparison between the 2 groups, we considered a difference in the CAL rate of 10 percentage points or less as clinically insignificant, and the noninferiority margin was set at 10%. As presented in Table 1, the overall CAL rate at 6 weeks was 15.9% (11 of 69 patients) in the IVIG plus aspirin group and 12.3% (8 of 65 patients) in the IVIG-alone group, with an overall difference of 3.6 percentage points (97.5% CI, −100.0 to 8.1 percentage points); the IVIG-alone group’s rate was 12.3% [3.6 percentage points] lower than the IVIG plus aspirin group. Separately, the CAL rate of LCA was 10.1% (7 of 69 patients) in the IVIG plus aspirin group and 7.7% (5 of 65 patients) in the IVIG-alone group, with an overall difference of 2.4 percentage points (97.5% CI, −100 to 7.1 percentage points); the IVIG-alone group’s rate was 7.7% [2.4 percentage points] lower than the IVIG plus aspirin group. The CAL rate of RCA was 7.7% (5 of 65 patients) in the IVIG-alone group and 5.8% (4 of 69 patients) in the IVIG plus aspirin group, with an overall difference of 1.9 percentage points (97.5% CI, −100.0 to 10.0 percentage points); the IVIG-alone group’s rate was 7.7% [1.9 percentage points] higher than the IVIG plus aspirin group. For the upper end of the CI overall, the LCA and RCA CAL rates were all below the noninferiority margin of 10%. In the current setting, for the test of noninferiority, the 1-sided *P* value would be .01, suggesting that we should reject this null hypothesis and conclude that the difference in the CAL rate was less than 10 percentage points. Therefore, the noninferiority was established in the current cohort size of patient numbers in both the IVIG plus aspirin group and the IVIG-alone group.

As shown in eTable 2 in Supplement 2 for the absolute value of artery diameters, the LCA significantly decreased across the study in both groups, starting at a mean (SD) 2.20 (0.44) mm and reducing to 2.02 (0.35) mm at 6 months (*P* < .001). The mean (SD) rate of reduction was similar between the IVIG plus aspirin group and the IVIG-alone group at each time point (baseline, 2.23 [0.48] mm vs 2.17 [0.39] mm, *P* = .90; 6 weeks, 2.05 [0.45] mm vs 1.99 [0.39] mm, *P* = .48; and 6 months, 1.97 [0.38] mm vs 2.07 mm [0.32], *P* = .10), and there was no significant interaction effect between time and groups (*P* = .17). Similarly, the left anterior descending artery exhibited a significant narrowing in both groups, starting at a mean (SD) 1.75 (0.42) mm and decreasing to 1.56 (0.38) mm at 6 months (*P* < .001). Once again, the mean (SD) pattern of reduction was comparable between the IVIG plus aspirin group and the IVIG-alone group (baseline, 1.74 [0.44] mm vs 1.76 [0.40] mm, *P* = .19; 6 weeks, 1.55 [0.39] mm vs 1.59 [0.36] mm, *P* = .58; and 6 months, 1.50 [0.39] mm vs 1.61 [0.36] mm, *P* = .14). However, no interaction effect was observed between time and groups (*P* = .68). Likewise, the RCA narrowed significantly over time, from a mean (SD) 1.91 (0.43) mm to 1.81 (0.36) mm at 6 months (*P* = .006), with no noticeable mean (SD) differences in the rate of reduction between the IVIG plus aspirin group and the IVIG-alone group (baseline, 1.87 [0.44] mm vs 1.95 [0.41] mm, *P* = .16; 6 weeks, 1.75 [0.41] mm vs 1.77 [0.39] mm, *P* = .75; and 6 months, 1.78 [0.37] mm vs 1.85 [0.34] mm, *P* = .29), and there was no significant interaction between time and RCA diameter in the groups (*P* = .83).

In addition to assessing CAL formation as the primary outcome, we also investigated IVIG resistance. eTable 3 in Supplement 2 indicates that both groups had 3 patients each with IVIG resistance. There were no significant differences in the rates of IVIG resistance between the groups.

### Prophylactic Effects on Coronary Artery Changes and CAL Frequency

Throughout the study duration, we noted that most cases of patients with CALs were detected during the acute phase. However, some patients initially had a normal coronary artery diameter during the acute phase but later developed CALs at 6 weeks or 6 months, even after receiving IVIG alone or IVIG plus aspirin treatment. To compare the treatment effect between the IVIG-alone group and the IVIG plus aspirin group, we conducted statistical analysis of CAL frequency exclusively on the patients with CALs identified during the acute phase.

For the treatment-effect comparison between the 2 groups, we focused on the patients with CALs before the acute-phase treatment and evaluated the number of recoveries from these patients after treatment. As depicted in Table 2 at the baseline, 13.0% (9 of 69 patients) in the IVIG plus aspirin group and 10.8% (7 of 65 patients) in the IVIG-alone group presented with CALs in at least 1 of 3 coronary arteries with an abnormal coronary artery diameter (*z* score >2.5 SD). At 6 months, all patients had recovered, and no significant differences were observed between the groups concerning the recovered coronary artery abnormalities during the study period. The percentage of CALs significantly decreased compared with baseline, starting from 6 weeks (1 of 65 [1.5%] in the IVIG-alone group and 1 of 69 [1.4%] in the IVIG plus aspirin group; *P* = .001) through the end of the study period at 6 months (0 for both groups; *P* < .001). Similarly, no significant differences in treatment effect were observed between the 2 groups on either the LCA or the RCA (detailed findings are described in eAppendix 2 in Supplement 2).

**Table 2.** Frequency of Abnormal Coronary Artery Diameter by CAL Type and Time Point: Treatment Effect^a^

CAL type	Time point								Overall *P* value^c^
	Baseline		At 6 wk			At 6 mo			
	IVIG alone (n = 65)	IVIG plus aspirin (n = 69)	IVIG alone (n = 65)	IVIG plus aspirin (n = 69)	*P* value^b^	IVIG alone (n = 65)	IVIG plus aspirin (n = 69)	*P* value^b^	
LCA	4 (6.2)	6 (8.7)	0	1 (1.4)	.006	0	0	.001	.23
LAD	0	0	0	0	NA	0	0	NA	NA
RCA	4 (6.2)	3 (4.3)	1 (1.5)	0	.07	0	0	.008	.18
Overall	7 (10.8)	9 (13.0)	1 (1.5)	1 (1.4)	.001	0	0	<.001	.42

Abbreviations: CAL, coronary artery lesion; IVIG, intravenous immunoglobulin; LAD, left anterior descending; LCA, main left coronary artery; NA, not applicable; RCA, right coronary artery.

^a^ Data are presented as No. (%) of patients.

^b^ CAL frequency difference compared with baseline.

^c^ CAL frequency changes between IVIG-alone and IVIG plus aspirin groups for the entire study period.

Following the treatment effect, we investigated the prophylactic effect on the frequency of CALs in patients who developed CALs later after IVIG-alone or IVIG plus aspirin treatment. We performed the same statistical analysis for the comparison of CAL frequency, as illustrated in eTable 4 in Supplement 2. No statistical difference was identified between the IVIG plus aspirin group and the IVIG-alone group. However, a minimal number of newly developed cases of CALs in patients were observed after treatment, including 1 in the IVIG plus aspirin group and 2 in the IVIG-alone group.

### KD Cluster Subgroup Analysis of C**AL Fr**equency

The study by Wang et al,^18^ which examined the heterogeneity of KD, identified 4 distinct patient clusters based on subjective clinical features and laboratory results: the liver subgroup, band subgroup, node subgroup, and young subgroup. To assess the impact of high-dose aspirin treatment on CAL formation within these diverse clusters, we further classified patients into 4 clusters in the 2 study groups. We conducted a comparative analysis of the overall CAL rates at baseline, 6 weeks, and 6 months across the 4 identified clusters and found that discernible trends of reduced CAL rates were evident in all 4 clusters at both 6 weeks and 6 months compared with baseline; however, no significant differences between the 2 groups were observed within the liver, node, or young clusters during the study periods (eTable 5 in Supplement 2).

## Discussion

This study represents, to our knowledge, the first multicenter randomized clinical trial investigating the efficacy of high-dose aspirin and IVIG during the acute stage of KD. We assessed the impact of discontinuing high-dose aspirin (80-100 mg/kg per day) on the occurrence of CALs during the acute-phase treatment of KD. The findings revealed that the elimination of high-dose aspirin did not yield a significant effect on CAL incidence. No significant differences were observed between the groups in terms of the frequency of abnormal coronary artery abnormalities during the study period. Through a statistical noninferiority comparison, we found that the CAL rate in the IVIG-alone group was not higher by 10% than that in the IVIG plus aspirin group, establishing noninferiority with clinical insignificance.

Preventing CAL formation is crucial, as it represents the most severe complication in KD. Therefore, determining the ideal aspirin dosage to prevent this complication is of paramount importance. Aspirin, a salicylate drug, works by inhibiting the production of prostaglandins, which are involved in the inflammatory response. By exerting anti-inflammatory and antiplatelet effects, aspirin is thought to play a role in suppressing the inflammatory response and reducing the risk of blood clots in KD.

Compared with high-dose aspirin, a previous study by investigators of our team demonstrated that the use of low-dose aspirin in the initial treatment of children with KD was not linked to fever recurrence or the formation of CALs.^8^ Additionally, administering high-dose aspirin during the acute stage of KD did not yield any benefits in terms of inflammation or improved treatment outcomes.

Evaluating the overall effectiveness of IVIG treatment entails considering both its treatment effect (the number of patients successfully treated) and prevention effect (the prevention of new cases of CALs in patients). Our analysis revealed no statistically significant differences in either the treatment effect or prophylactic effect between these 2 groups, despite the occurrence of very limited newly developed cases of CALs in patients. Conclusions regarding the prevention effect may require a larger cohort to ensure robust findings.

Furthermore, we performed an analysis to compare the effectiveness in 4 subgroups of KD, which were categorized based on subjective clinical features and laboratory results. Within each of these individual clusters or subgroups, no significant difference was observed in the reduction of CAL rates at 6 weeks or 6 months after treatment during the acute phase between the IVIG plus aspirin group and the IVIG-alone group (detailed results are described in eTables 5-10 in Supplement 2).

The primary purpose of administering high-dose aspirin was to elicit anti-inflammatory effects. As a result, we conducted an analysis comparing efficacy in subgroups classified based on the acute-phase levels of 2 inflammatory biomarkers: C-reactive protein and platelet count (detailed results are described in eTables 11-14 in Supplement 2). Consistent with findings in other subgroups, we identified no significant distinction between the IVIG plus aspirin group and the IVIG-alone group in either subgroup in the low (normal) range or in the high (abnormal) range for C-reactive protein or platelet count. The analysis of these subgroups indicates that the administration of IVIG treatment alone, without the addition of high-dose aspirin, did not increase the risk of CAL formation 6 weeks or later after the initial treatment. This approach has wider relevance for patients with KD with varied demographic and clinical characteristics.

### Limitations

This study has some limitations. First, the participants only represented an East Asian population. Another limitation is the relatively small sample size and a limited number of patients with CALs in both the IVIG plus aspirin group and the IVIG-alone group. Collecting additional data points should contribute to narrowing the CI for the difference, allowing for a more stringent assessment of noninferiority between the 2 groups. Furthermore, we exclusively enrolled patients with typical KD in the current trial. Future studies focusing on patients with atypical KD would be valuable to determine whether the conclusion regarding the equivalent efficacy of IVIG alone in preventing CAL formation remains valid.

## Conclusions

In this randomized clinical trial of IVIG-only treatment for CALs among children with KD, high-dose aspirin did not play a significant role in managing CALs in patients with KD. Administering the standard 2 g/kg per day IVIG without high-dose aspirin (80-100 mg/kg per day) during the acute-phase therapy for KD did not increase the risk of CALs, which are a primary cause of morbidity and mortality in patients with KD. Results from a comparison analysis indicated the noninferiority between the IVIG-alone group and the IVIG plus aspirin group. Therefore, addition of high-dose aspirin during initial IVIG treatment was not statistically significant or clinically meaningful. Future studies or similarly designed clinical trials in other race and ethnicity or geographic populations may be necessary to confirm the broad applicability of our findings.

## Notes

Supplement 1.
Trial Protocol

Supplement 2.
eMethods
**eAppendix 1.** Clinical Trial Design and Baseline Characteristics **eAppendix 2.** Categorized Artery and KD Subgroup Analysis **eTable 1.** Baseline Characteristics and Group Distribution of Participants in the Study **eTable 2.** Analysis of Variance of the Clinical Parameters in Terms of the Group, Time, and Their Interaction **eTable 3.** Comparison of IVIG Treatment Resistance **eTable 4.** Prophylactic Effect: Comparison Frequency of Abnormal Coronary Arteries Diameter in Term of Groups Type and Time **eTable 5.** Comparison Frequency of Abnormal Coronary Arteries Diameter in Liver, Band, Node, and Young Age Clusters **eTable 6.** Comparison Frequency of Abnormal Coronary Arteries Diameter-Liver Cluster **eTable 7.** Comparison Frequency of Abnormal Coronary Arteries Diameter-Band Cluster **eTable 8.** Comparison Frequency of Abnormal Coronary Arteries Diameter-Node Cluster **eTable 9.** Comparison Frequency of Abnormal Coronary Arteries Diameter-Young Age Onset Cluster **eTable 10.** Comparison Frequency of Abnormal Coronary Arteries Diameter-Subgroup Populations From Liver, Band, Node, and Young Age Clusters **eTable 11.** Comparison Frequency of Abnormal Coronary Arteries Diameter-Low CRP Subgroup (CRP ≤ 100 mg/L) **eTable 12.** Comparison Frequency of Abnormal Coronary Arteries Diameter-High CRP Subgroup (CRP > 100 mg/L) **eTable 13.** Comparison Frequency of Abnormal Coronary Arteries Diameter-Low Platelet Count Subgroup (Platelet < 400 × 10^3^/μL) **eTable 14.** Comparison Frequency of Abnormal Coronary Arteries Diameter-High Platelet Count Subgroup (Platelet ≥ 400 × 10^3^/μL)
eReferences

Supplement 3.
Data Sharing Statement
