# Supplementary material for: Intravenous Immunoglobulin Alone for Coronary Artery Lesion Treatment of Kawasaki Disease: A Randomized Clinical Trial
Source: JAMA Netw Open. 2025 Apr 3;8(4):e253063. doi: 10.1001/jamanetworkopen.2025.3063 (PMC11969286; doi:10.1001/jamanetworkopen.2025.3063)
Supplement: Supplement 1. — Trial Protocol [file jamanetwopen-e253063-s001.pdf]

Protocol

This supplement contains the following items:

1. Original protocol, which was unchanged and only one version exists.
2. Original statistical analysis plan, which was unchanged and only one version exists

Study Title:

A multi-center, randomized, parallel-group, open-label, non-inferiority study to compare the efficacy of intravenous immunoglobulin alone and intravenous immunoglobulin plus high-dose aspirin in acute stage of Kawasaki disease for preventing coronary artery lesion (KIAA trial)

**Trial Registration:**	ClinicalTrials.gov: NCT02951234
**Protocol Number:**	KCGCTC-001
**Version Number:**	1.0
**Version Date:**	20160620
**Funding:**	CPRP
**Principal Investigators:**	Ho-Chang Kuo, MD, PhD
**Protocol Authors:**	Ho-Chang Kuo, MD, PhD; Man-Jen Hsu, PhD

Table of Contents

Table of Contents 3

Protocol Synopsis 5

List of abbreviations 8

1. Administrative information 9

1.1 Title 9

1.2 Trial registration 9

1.3 Protocol version 9

1.4 Funding 9

1.5 Roles and responsibilities 9

2. Introduction 10

2.1 Background and rationale 10

2.2 Objectives 10

2.3 Trial design 10

3. Methods 11

3.1 Study setting 12

3.2 Eligibility criteria 12

3.3 Interventions 15

3.4 Outcomes 16

3.5 Participant timeline 18

3.6 Sample size 19

3.7 Recruitment 20

3.8 Allocation 20

3.9 Blinding 20

3.10 Data collection methods and management 20

3.11 Statistical methods 20

3.12 Adverse events and serious adverse events 21

3.13 Auditing 22

4. Ethics and dissemination 23

4.1 Research ethics approval 23

4.2 Protocol amendments………...**23**
4.3 Consent or assent………...**23**
4.4 Confidentiality…………..**23**
4.5 Dissemination policy…………..**24**
References…………...25

Appendices………...27

Protocol Synopsis

Title:

A multi-center, randomized, parallel-group, open-label, non-inferiority study to compare the efficacy of intravenous immunoglobulin alone and intravenous immunoglobulin plus high-dose aspirin in acute stage of Kawasaki disease for preventing coronary artery lesion (KIAA trial)

Objectives:

Primary objective is to assess whether intravenous immunoglobulin (IVIG) alone as the primary therapy in acute stage of Kawasaki disease (KD) is non-inferior to IVIG plus high-dose aspirin therapy in preventing the development of coronary artery lesion (CAL) at 6-8 weeks.

Secondary objectives are to compare IVIG resistance rate, duration of fever after IVIG, duration of hospitalization, laboratory data, Z score of coronary artery, CAL at 6 months, adverse event (AE) and serious adverse event (SAE) between IVIG alone and IVIG plus high-dose aspirin as the primary therapy in acute stage of KD.

Eligibility:

Inclusion criteria

1. Male or female, age less than 6 years old
2. Fulfilled the AHA criteria for KD as below:

(1) Fever (more than 38.0℃ear temperature) ≧ 5 days, and 4 of the 5 following symptoms

(2) Diffuse mucosal inflammation (strawberry tongue, dry and fissured lips)

(3) Bilateral non-purulent conjunctivitis

(4) Dysmorphous skin rashes

(5) Indurative edematous change over the hands and feet, or desquamation over the finger or toe tip

(6) Cervical lymphadenopathy (one or more nodule at least 1.5 cm in diameter)

3. Informed consent form (ICF) signed by the patient or a legal guardian

Exclusion criteria

1. Symptoms not full fit the KD criteria

2. Acute fever for ≦ 5 days or >10 days

3. IVIG treatment at other hospital before refers to study site

4. Treatment with corticosteroids, other than inhaled forms, in the previous 2 weeks

5. Presence of a disease known to mimic Kawasaki disease (such as systemic juvenile idiopathic arthritis, Steven-Johnson Syndrome, Macrophage activation syndrome)

6. Previous diagnosis of KD

7. Inability to take aspirin (history of hypersensitivity to aspirin, G6PD deficiency, intolerance to aspirin)

8. Inability to take IVIG (history of allergic reactions for IVIG, allergic reactions when testing dose of IVIG, selective IgA deficiency)

9. Afebrile before enrolment

10. Concomitant severe medical disorders (e.g. immunodeficiency, chromosomal anomalies, congenital heart diseases, metabolic diseases, nephritis, collagen diseases)

11. Suspected severe infectious disease (e.g. sepsis, septic meningitis, peritonitis, bacterial pneumonia, varicella, and influenza)

12. Conditions judged by the investigator as unsuitable for this trial

Study treatment:

Test group: All patients will receive IVIG (2g/kg) in 10-12 hours alone, **<u>without</u>** high-dose aspirin. After fever subsides, low-dose aspirin (3-5mg/kg/day) will be prescribed until 6-8 weeks.

Standard group: All patients will receive IVIG (2g/kg) in 10-12 hours plus high-dose aspirin (80-100mg/kg/day, divided into four doses) till fever subside. After fever subsides, low-dose aspirin (3-5mg/kg/day) will be prescribed until 6-8 weeks.

Primary endpoint:

The primary endpoint is CAL rate at 6-8 weeks.

Sample size estimation:

A total of 278 patients will be recruited to achieve 250 per-protocol (PP) patients.

List of abbreviations

AE	Adverse event
AHA	American Heart Association
CAL	Coronary artery lesion
CRF	Case report form
CRP	C-reactive protein
CTCAE	Common Terminology Criteria for Adverse Events
G6PD	glucose-6-phosphate dehydrogenase
GCP	Good clinical practice
ICF	Informed consent form
IRB	Institutional Review Board
ITT	Intention-to-treat
IVIG	intravenous immunoglobulin
KD	Kawasaki disease
LAD	Left anterior descending artery
LMCA	Left main coronary artery
PP	Per-protocol
RCA	Right coronary artery
RCT	Randomized controlled trial
SAE	Serious adverse event

1. Administrative information

1.1 Title

A multi-center, randomized, parallel-group, open-label, non-inferiority study to compare the efficacy of intravenous immunoglobulin alone and intravenous immunoglobulin plus high-dose aspirin in acute stage of Kawasaki disease for preventing coronary artery lesion (KIAA trial)

1.2 Trial registration

The clinical trial will be registered on ClinicalTrials.gov

1.3 Protocol version

Protocol Number: KCGCTC-001

Version Number: 1.0

Version Date: 20160620

1.4 Funding

An application for funding to support this trial will be made to Chang Gung Memorial Hospital (CPRP).

1.5 Role and responsibilities

Principal Investigator (PI): Ho-Chang Kuo, M.D., Ph.D.

No.123 Dapi Rd. Niaosong Dist., Kaohsiung City 83301, Taiwan

Email: erickuo48@yahoo.com.tw

Clinical research coordinator (CRC): May-Gung Chen

Email: chen.mico61@gmail.com

2. Introduction

2.1 Background and rationale

Kawasaki disease (KD) is an acute febrile systemic vasculitis that was described by Kawasaki et al. in 1974.[1] In developed countries, it is the leading cause of acquired heart diseases in children.[2-3] It mainly affects children younger than five years old, especially in Asian countries. Reported incidence rates in Taiwan, Japan, and Korea ranges from 69 to 213 cases per 100,000 children under the age of five years.[4-6] The most serious complication of KD is the occurrence of coronary artery lesions (CAL), including coronary artery dilatation, coronary artery aneurysm, and coronary artery fistula formation, which may lead to myocardial infarction, sudden death, or ischemic heart disease.[7-8] In our previous published data, 35 of 341 (10.3%) Taiwanese children had CAL formation 8 weeks after disease onset.[9] Although the clinical features of KD are recognizable, its underlying immuno-pathogenetic mechanisms are still under investigation, particularly the culprit for CAL development.[10-12]

Aspirin has been used in the treatment of KD for many years even before the advent of intravenous immunoglobulin (IVIG). Standard treatment for acute phase of KD introduced by American Heart Association (AHA) and American Academy of Pediatrics (AAP) includes IVIG therapy with a single dose of 2 g/kg infusion over 10-12 h plus oral administration of aspirin at high-dose of 80-100 mg/kg/d.[7] Although aspirin has important anti-inflammatory (high-dose) and anti-platelet (low-dose) effects, no prospective study has confirmed that aspirin reduced the incidence of CAL formation. Some reports have shown that the incidence of CAL is highly dependent on the dose of IVIG, but independent of aspirin dose.[13,14] This suggests that only anti-platelet aspirin dose may be adequate during the acute phase of KD. In Taiwanese data, Hsieh et al. reported that high-dose aspirin in the acute stage of KD has no effect on the response rate of IVIG therapy, duration of fever, or incidence of CAL when children are treated with high-dose (2 g/kg) IVIG as a single infusion, despite treatment before or after day 5 of illness.[15] Our recent retrospective study investigated 851 KD patients from two medical centers in Taiwan (Chang Gung Memorial Hospital-Kaohsiung and Kaohsiung Veterans General Hospital).[16] The children were divided into Group 1, IVIG with high-dose aspirin

(n=305) and Group 2, IVIG without high-dose aspirin (n=546). There are no significant differences between groups 1 and 2 in terms of gender (p=0.51), IVIG resistant rate (31/305 vs. 38/546, p=0.07), CAL formation rate (52/305 vs. 84/546, p=0.67), and total hospital days (6.3±0.2 vs. 6.7±0.2, p=0.13). These retrospective observational data suggest that aspirin offers little additional benefit to IVIG therapy.

There is still no good quality of randomized control trial (RCT) to evaluate whether children with KD should continue to receive high-dose aspirin as part of their treatment regimen. Cochrane Vascular Group reviewed RCTs of aspirin to treat KD in children.[17] The review authors identified only one RCT (multicenter, 3-arms design), from Japan reported in 1991. Furusho et al compared IVIG and aspirin treatment with aspirin alone, and with IVIG alone.[18] The dose of aspirin was 30 to 50 mg/kg/day in three divided doses until the fever had subsided, then 10 to 30mg/kg/day once a day until “the acute reaction had also disappeared.” Children were enrolled if they presented within seven days of the onset of symptoms. Children with recurrent KD and with CAL at presentation were excluded. Children in the two groups (IVIG with aspirin and IVIG alone) did not differ significantly in respect to age at presentation, sex, or time from onset of symptoms to trial entry. The incidence of new CAL prior to 30 days from onset of symptoms were 18.4% (9 of 49) in children receiving IVIG and aspirin and 18.9% (10 of 53) in children receiving IVIG alone. The relative risk (RR) was 0.97 and 95% confidence intervals (CI) was 0.43 to 2.19. The prevalence of CAL at 30 days after the onset of symptoms were 10.2% (5 of 49) in children receiving IVIG and aspirin and 7.8% (4 of 51) in children receiving IVIG alone. The RR was 1.30 and 95% CI was 0.37 to 4.56.

The risk of aspirin therapy in children with KD is considered to be low and appear to be similar to those reported in other settings. However, serious aspirin side effects have been reported in children treated for KD, including hepatic toxicity, gastritis, upper gastrointestinal bleeding, sensorineural hearing loss, and rarely, Reye’s syndrome.[19-22] Considering the risks of drug toxicity and the lack of evidence for preventing CAL formation, the role of high-dose aspirin in the acute phase of KD needs to be reassessed. Therefore, we initiate this multi-center, prospective, parallel-group, open-label, non-

inferiority RCT to investigate whether high-dose aspirin in the acute stage of KD has beneficial effects on the prevention of CAL formation.

2.2 Objectives

Primary objective

- To assess whether IVIG alone as the primary therapy in acute stage of KD is non-inferior to IVIG plus high-dose aspirin therapy in preventing the development of CAL at 6-8weeks.

Secondary objectives

- To compare IVIG resistance rate, duration of fever after IVIG, duration of hospitalization, laboratory data (CBC/DC, GOT/GPT, and C-reactive protein (CRP)), Z score of coronary arteries, CAL at 6 months, adverse event (AE) and serious adverse event (SAE) between IVIG alone and IVIG plus high-dose aspirin as the primary therapy in acute stage of KD.

2.3 Trial design

The trial is designed as a multi-center, prospective, randomized controlled, open-label, non-inferiority trial with two parallel groups to assess the efficacy of IVIG alone for the primary treatment in acute stage of KD. The primary endpoint is defined as the CAL formation at 6-8 weeks. Patients who meet the eligibility criteria are randomly assigned (1:1) to a test group (receiving IVIG alone) or a standard group (receiving IVIG plus high dose aspirin).

3. Methods

3.1 Study setting

This clinical trial will be conducted at 7 medical centers in Taiwan, including:

Site 1: Kaohsiung Chang Gung Memorial Hospital (KCGMH)

Site 2: Linkou Chang Gung Memorial Hospital (LCGMH)

Site 3: National Taiwan University Hospital (NTUH)

Site 4: Mackay Memorial Hospital (MKMH)

Site 5: Taichung Veterans General Hospital (TVGH)

Site 6: China Medical University Hospital (CMUH)

Site 7: Kaohsiung Veterans General Hospital (KVGH)

3.2 Eligibility criteria

Eligible patients are those who meet all of the inclusion criteria mentioned below and none of the listed exclusion criteria:

Inclusion criteria

Patients eligible for the clinical trial must comply with all of the following at randomization.

1. Male or female, age less than 6 years old

2. Fulfilled the AHA criteria for KD as below:

   (1) Fever (more than 38.0℃ear temperature) ≧ 5 days, and 4 of the 5 following symptoms

   (2) Diffuse mucosal inflammation (strawberry tongue, dry and fissured lips)

   (3) Bilateral non-purulent conjunctivitis

(4) Dysmorphous skin rashes

(5) Indurative edematous change over the hands and feet, or desquamation over the finger or toe tip

(6) Cervical lymphadenopathy (one or more nodule at least 1.5 cm in diameter)

3. Informed consent form (ICF) signed by the patient or a legal guardian

Exclusion criteria

Patients fulfilling any of the following criteria are not eligible for inclusion in this clinical trial.

1. Symptoms not full fit the KD criteria
2. Acute fever for ≦ 5 days or >10 days
3. IVIG treatment at other hospital before refers to study site
4. Treatment with corticosteroids, other than inhaled forms, in the previous 2 weeks
5. Presence of a disease known to mimic Kawasaki disease (such as systemic juvenile idiopathic arthritis, Steven-Johnson Syndrome, Macrophage activation syndrome)
6. Previous diagnosis of KD
7. Inability to take aspirin (history of hypersensitivity to aspirin, G6PD deficiency, intolerance to aspirin)
8. Inability to take IVIG (history of allergic reactions for IVIG, allergic reactions when testing dose of IVIG, selective IgA deficiency)
9. Afebrile before enrolment
10. Concomitant severe medical disorders (e.g. immunodeficiency, chromosomal anomalies, congenital heart diseases, metabolic diseases, nephritis, collagen diseases)
11. Suspected severe infectious disease (e.g. sepsis, septic meningitis, peritonitis, bacterial pneumonia, varicella, and influenza)
12. Conditions judged by the investigator as unsuitable for this trial

Withdraw criteria

Participants should be withdrawn from the clinical trial if any of the conditions set below has occurred. The reason for a patient withdrawn from the study will be recorded in the case report form (CRF) and in the participant's medical record.

1. Participant or his/her legal guardian decides to withdraw his/her ICF.

2. Participant has allergic reaction during IVIG or aspirin treatment.

3. Participant develops AE or SAE that the investigator considered a cessation of the study treatment is necessary.

4. Participant is lost of follow up.

5. Investigator considers that the participant is no longer physically and/or psychologically feasible to remain in the study.

3.3 Intervention

- **Test group**

All patients will receive IVIG (2g/kg) in 10-12 hours alone, **without** high-dose aspirin. After fever subsides, low-dose aspirin (3-5mg/kg/day) will be prescribed until 6-8 weeks.

- **Standard group**

All patients will receive IVIG (2g/kg) in 10-12 hours plus high-dose aspirin (80-100mg/kg/day, divided into four doses) till fever subside. After fever subsides, low-dose aspirin (3-5mg/kg/day) will be prescribed until 6-8 weeks.

- **Concomitant treatment**

The investigators should try to minimize the concomitant treatments for the patient during the study. Any use of concomitant treatment must be recorded on the CRF.

- **Prohibited treatment**

Use of steroid, anti-TNF, anti-IL6, anti-IL17, anti-CD20 (etc. biologic agents) is not allowed during the study treatment unless the subject has withdrawn from the trial.

3.4 Outcomes

Primary outcome

- **CAL**

  The primary outcome is CAL at 6-8 weeks.

CAL will be evaluated by experienced pediatric cardiologists (at least 2 years experience of pediatric 2D echocardiography) using 2D echocardiography. The luminal diameter of left main coronary artery (LMCA) is measured at a point between the ostium and the first bifurcation of the artery (as Figure below). The luminal diameter of left anterior descending artery (LAD) is measured distal to and away from the bifurcation of the branch from the LCA. The luminal diameter of right coronary artery (RCA) is measured in the relatively straight section of the artery just after rightward turn from the initial anterior course of the artery. Definition of CAL is coronary artery luminal diameter greater than +3SD in one of the three proximal segments or greater than +2.5SD in two of the three proximal segments. The calculation of Z score is based on the website of Taiwan Society of Pediatric Cardiology (http://www.tspc.org.tw/service/z_score.asp).

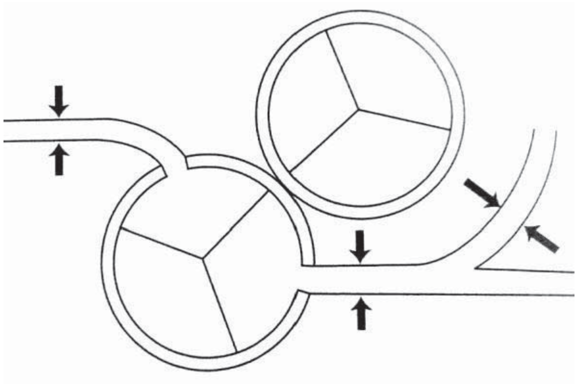

Figure: coronary artery luminal diameter measurement in 2D echocardiogram

Secondary outcomes

- **IVIG resistance rate:**

IVIG resistance is defined as persistent or recrudescent fever at least 48 hours but not longer than 7 days after completion of the first IVIG treatment.

- **Duration of fever after IVIG treatment**

  We will record the body temperature every 6 hour during the hospitalization period.

- **Duration of hospitalization**

  We will record the hospitalization duration from admission (or arriving emergency room) to discharge date.

- **Laboratory data**

  Laboratory data of CBC/DC, GOT/GPT, and CRP will be checked at baseline, day 5, and 6-8 week.

- **Z score of coronary arteries**

  We will estimate Z score of the proximal segments of LMCA, LAD, and RCA at baseline, Day 5, 6-8 weeks, and 6 month with 2D echocardiography. The calculation of Z score is based on the website of Taiwan Society of Pediatric Cardiology (http://www.tspc.org.tw/service/z_score.asp).

- **CAL rate at 6 months**
- **Adverse event (AE) and serious adverse event (SAE)**

  We will record AE and SAE. Severity of AE will be based on Common Terminology Criteria for Adverse Events (CTCAE) version 4.03.

3.5 Participant timeline

**Study period**	Enrolment	Randomization	Intervention	Day 5	6-8 weeks	6 months
**Time point**	Day -3~0		Day 1	Day 5±2	Day 49±10	Day 180±30
Eligibility screen	●					
Informed consent	●					
Demographics	●					
Randomization		●				
Intervention			●			
echocardiography				●	●	●
Body temperature	●			●	●	
CBC/DC	●			●	●	
GOT/GPT	●			●	●	
CRP	●			●	●	
AE、SAE			●	●	●	

3.6 Sample size

The sample size determination is based on the result of our retrospective data.[16] The estimated proportions of CAL are 17% after IVIG and high-dose aspirin combination treatment and 15.4% after IVIG alone treatment. We consider the difference less than 10% is of no clinical importance. The following contents are the statistical basis for estimating sample size for this trial.

H_0: $P_T - P_S \leqq \delta$

H_A: $P_T - P_S > \delta$

P_T: CAL-free rate in test group =0.846

P_S: CAL-free rate in standard group =0.83

δ= -0.1

Assume that the type I and type II error rates are $\alpha = 0.05$, $\beta = 0.2$, respectively. The clinically meaningful difference δ is -0.1. The CAL-free rate of test group (IVIG alone) P_T is 0.846 and that of standard group (IVIG + high dose aspirin) P_S is 0.83.

Assume the sample sizes of two groups are equal, the sample size of each group for the non-inferiority trial is125 patients. Offsetting a maximal dropout of 10%, 139 patients are required for per group. That is, a total sample size of 278 patients is required for the trial.

3.7 Recruitment

The clinical trial will be declared and registered at the ClinicalTrial.gov. Recruitment will be start after the protocol approved by the Institutional Review Board (IRB) at each sites of the participating institutions and will be ended in 2018 or until a total of 278 participants have been recruited.

3.8 Allocation

Eligible participants will be randomized to either the IVIG alone or IVIG plus high-dose aspirin on an equal ratio. The investigator or his/her delegate will contact the Clinical Trial Center of Kaohsiung Chang Gung Memorial Hospital (KCGMH- CTC) after confirming that the patient fulfills all the inclusion/exclusion criteria. The KCGMH- CTC will assign a treatment arm to the patient. Randomization will use permuted blocks of random sizes. The block size will not be disclosed to ensure concealment.

3.9 Blinding

Participants and study investigators are unblinded to the group of IVIG alone or IVIG plus high-dose aspirin.

3.10 Data collection methods and management

All data including demographic data, medical history, medical record, laboratory data, 2D echocardiography, AE, and SAE will be recorded at CRF. The investigators will maintain individual records (ICF and CRF) for each patient as source data. A copy of each CRF will be sent to KCGMH-CTC for data management.

3.11 Statistical methods

All baseline characteristics will be used to investigate comparability of both trial arms. The primary outcome evaluation (CAL rate at 6-8 weeks) and secondary outcome of Z score of coronary arteries and CAL rate at 6 months will be performed on per-

protocol (PP) population while the other secondary outcome will be performed on intention-to-treat (ITT) population. Continuous variables will be described by using the mean (standard deviation) or median (interquartile range) as appropriate, categorical variables by using count (percentage). The participant characteristics will be compared using a χ^2 test for categorical variables, and a t test or Wilcoxon rank sum test for continuous variables. P values<0.05 will be considered statistically significant.

3.12 Adverse Events (AE) and Serious Adverse Events (SAE)

- **Adverse Events**

An AE is any untoward medical occurrence in a subject during the participation of a clinical trial, regardless the possibility of a causal relationship. An AE can be any unfavorable signs or symptoms or diagnosed new diseases or deterioration of existing chronic or intermittent diseases. AEs will be collected after the participant has provided consent and enrolled in this clinical trial. If a subject experiences an AE after the informed consent document is signed but the subject has not started to receive treatment, the event will be reported as not related to the clinical trial.

- **Serious Adverse Events**

An SAE for this study is any untoward medical occurrence that is believed by the investigators to be causally related to treatment of the clinical trial and results in any of the following:

(1) Results in death

(2) Life-threatening, meaning the subject is at risk of death at the time of event.

(3) Requires hospitalization or prolongation of existing hospitalization. Emergency room visits that do not result in admission to the hospital should not be considered as a serious adverse event of requiring hospitalization or prolongation of existing hospitalization, and such emergency room visits should be evaluated for one of the other serious outcomes instead.

(4) Results in disability/incapacity.

(5) Is a congenital anomaly/birth defect.

(6) Events requiring medical and/or surgical intervention to prevent one of the other outcomes listed in the definition above or, based on medical and/or scientific judgment of investigator, are important and should be considered serious.

- **Disease-related Event**

The disease-related event is defined as an event that can be explained by the nature cause of KD, including eye redness, skin rash, and desquamation. The disease-related events and conditions are usually recorded according to the protocol and should not be considered as an AE. For those events that the investigator considers to be more severe than expected, should be reported as AEs or SAEs as appropriate. For all events that result in death or are life-threatening, must be reported SAEs.

- **Reporting and Recording of AE and SAE**

All AEs occurring after entry into the study and until hospital discharge will be recorded. All AEs and SAEs should be documented in the source documents and the relevant CRF and SAE form when applicable. The investigator may be asked to provide photocopies of the medical records for completing the AE or SAE report. It is the responsibility of the investigator to report AEs or SAEs by diagnosis terminologies, if possible. The investigator will be asked to determine the severity and causality of each AE and SAE based on the CTCAE version 4.03 and investigator's clinical judgment.

3.13 Auditing

This trial will be audited by independent reviewers for the participant enrolment, consent, eligibility, allocation to study groups and policies to protect participants, including reporting of AE and SAE based on regulation of each participating institutions.

4. Ethics and dissemination

4.1 Research ethics approval

The trial will be approved by the Institutional Review Board (IRB) at each of the participating institutions and will be conducted in accordance with GCP standards.

4.2 Protocol amendments

Any modifications to the protocol which may impact on the conduct of the study, potential benefit of the patient or may affect patient safety, including changes of study objectives, study design, patient population, sample sizes, study procedures, or significant administrative aspects will require a formal amendment to the protocol. Such amendment will be agreed upon by the IRB prior to implementation, except in the case of changes made to protect patient safety, which will be implemented immediately.

4.3 Consent or assent

Investigators or trained research nurses will introduce the trial to potential participants and their legal guardians regarding the main aspects of the trial. All participants and their legal guardians will receive adequate information about the nature, purpose, possible risks and benefits of the trial, and about alternative therapeutic choices, using an ICF approved by the IRB. The participants and their legal guardians will be given ample time and opportunity to ask questions and to consider participation in the trial. The ICF, signed by the participant and their legal guardians, is required for enrolment in the trial. The investigators will maintain the original and a copy of the signed consent form with the trial records.

4.4 Confidentiality

All study related information will be stored securely at each study site. All participant information will be stored in locked file cabinets in areas with limited access.

To assure confidentiality, participants will be allocated a unique trial identification number throughout the trial. All records that contain names or other personal identifiers will be stored separately from study records identified by code number.

4.5 Dissemination policy

The protocol will be declared in a publicly accessible database (ClinicalTrials.gov). The trial will close out after the last participant's follow up data has been completed. The trial result will be disseminated though peer-reviews publications and conference presentations.

References

1. Kawasaki T, Kosaki F, Okawa S, Shigematsu I, Yanagawa H. A new infantile acute febrile mucocutaneous lymph node syndrome (MLNS) prevailing in Japan. Pediatrics 1974;54(3):271-6.
2. Wang CL, Wu YT, Liu CA, Kuo HC, Yang KD. Kawasaki disease: infection, immunity and genetics. Pediatr Infect Dis J 2005;24(11):998-1004.
3. Burns JC, Glode MP. Kawasaki syndrome. Lancet 2004;364(9433):533-44.
4. Huang WC, Huang LM, Chang IS, Chang LY, Chiang BL, Chen PJ, et al. Epidemiologic features of Kawasaki disease in Taiwan, 2003-2006. Pediatrics 2009;123(3):e401-5.
5. Nakamura Y, Yashiro M, Uehara R, Oki I, Kayaba K, Yanagawa H. Increasing incidence of Kawasaki disease in Japan: nationwide survey. Pediatr Int 2008;50(3):287-90.
6. Park YW, Han JW, Park IS, Kim CH, Cha SH, Ma JS, et al. Kawasaki disease in Korea, 2003-2005. Pediatr Infect Dis J 2007;26(9):821-3.
7. Newburger JW, Takahashi M, Gerber MA, Gewitz MH, Tani LY, Burns JC, et al. Diagnosis, treatment, and long-term management of Kawasaki disease: a statement for health professionals from the Committee on Rheumatic Fever, Endocarditis and Kawasaki Disease, Council on Cardiovascular Disease in the Young, American Heart Association. Circulation 2004;110(17):2747-71.
8. Liang CD, Kuo HC, Yang KD, Wang CL, Ko SF. Coronary artery fistula associated with Kawasaki disease. Am Heart J 2009;157(3):584-8.
9. Kuo HC, Yu HR, Juo SH, Yang KD, Wang YS, Liang CD, et al. CASP3 gene single-nucleotide polymorphism (rs72689236) and Kawasaki disease in Taiwanese children. J Hum Genet 2011;56(2):161-5.
10. Kuo HC, Wang CL, Liang CD, Yu HR, Chen HH, Wang L, et al. Persistent monocytosis after intravenous immunoglobulin therapy correlated with the development of coronary artery lesions in patients with Kawasaki disease. J Microbiol Immunol Infect 2007;40(5):395-400.
11. Kuo HC, Wang CL, Liang CD, Yu HR, Huang CF, Wang L, et al. Association of lower eosinophil-related T helper 2 (Th2) cytokines with coronary artery lesions in Kawasaki disease. Pediatr Allergy Immunol 2009;20(3):266-72.
12. Weng KP, Hsieh KS, Hwang YT, Huang SH, Lai TJ, Yuh YS, et al. IL-10 polymorphisms are associated with coronary artery lesions in acute stage of Kawasaki disease. Circ J 2010;74(5):983-9.

13. Durongpisitkul K, Guruaj VJ, Park JM, Marin CF. The prevention of coronary artery aneurysm in Kawasaki disease: a meta-analysis on the efficacy of aspirin and immunoglobulin treatment. Pediatrics 1995; 96(6):1057–61.

14. Terai M, Shulman ST. Prevalence of coronary artery abnormalities in Kawasaki disease is highly dependent on gamma globulin dose but independent of salicylate dose. J Pediatr 1997; 131(6):888–93.

15. Hsieh KS, Weng KP, Lin CC, Huang TC, Lee CL, Huang SM. Treatment of acute Kawasaki disease: aspirin's role in the febrile stage revisited. Pediatrics 2004;114(6):e689-93.

16. Kuo HC, Lo MH, Hsieh KS, Guo MM, Huang YH. High-Dose Aspirin Is Associated with Anemia and Does Not Confer Benefit to Disease Outcomes in Kawasaki Disease. PloS one. 2015;10(12):e0144603. Epub 2015/12/15.

17. Baumer JH, Love SJ, Gupta A, Haines LC, Maconochie I, Dua JS. Salicylate for the treatment of Kawasaki disease in children. Cochrane Database Sys Rev. 2006; 18(4):CD004175.

18. Furusho K, Kamiya T, Nakano H, Kiyosawa N, Shinomiya K, Hayashidera T et al. Intravenous gamma-globulin for Kawasaki disease. Acta Paediatr Jpn. 1991; 33(6):799-804.

19. Matsubara T, Mason W, Kashani IA, Kligerman M, Burns JC. Gastrointestinal hemorrhage complicating aspirin therapy in acute Kawasaki disease. *J Pediatr* 1996;128(5 Pt 1):701-3.

20. Chen CH, Lin LY, Yang KD, Hsieh KS, Kuo HC. Kawasaki disease with G6PD deficiency- report of one case and literature review. J Microbiol Immunol Infect. 2014; 47(3):261-263.

21. Sundel RP, Newburger JW, McGill T, et al. Sensorineural hearing loss associated with Kawasaki disease. *J Pediatr* 1990;117:371-7.

22. Wei CM, Chen HL, Lee PI, Chen CM, Ma CY, Hwu WL. Reye’s syndrome developing in an infant on treatment of Kawasaki syndrome. J Paediatr Child Health. 2005; 41(5-6):303-304.

Appendices

Appendix A

Principal Investigator’s Signature Page (Site 1 KCGMH)

Co- Investigator’s Signature Page (Site 1 KCGMH)

Principal Investigator’s Signature Page (Site 2 LCGMH)

Co- Investigator’s Signature Page (Site 2 LCGMH)

Principal Investigator’s Signature Page (Site 3 NTUH)

Co- Investigator’s Signature Page (Site 3 NTUH)

Principal Investigator’s Signature Page (Site 4 MKMH)

Co- Investigator’s Signature Page (Site 4 MKMH)

Principal Investigator’s Signature Page (Site 5 TVGH)

Co- Investigator’s Signature Page (Site 5 TVGH)

Principal Investigator’s Signature Page (Site 6 CMUH)

Co- Investigator’s Signature Page (Site 6 CMUH)

Principal Investigator’s Signature Page (Site 7 KVGH)

Co- Investigator’s Signature Page (Site 7 KVGH)

Appendix B: Case Report Form

Appendix C: Common Terminology Criteria for Adverse Events (CTCAE) version 4.03. (A complete copy of the CTCAE version 4.03 will be held in each site study file)

Statistical analysis

All of the baseline characteristics are used to study the comparability of both trial arms. The primary outcome evaluation and safety evaluation are carried out on the intention-to-treat (ITT) population. Continuous variables will be described using either the mean (standard deviation) or median (interquartile range), as appropriate, and categorical variables will use count (percentage). Participants' characteristics are compared using a $\chi 2$ test for categorical variables and a t-test or Wilcoxon rank sum test for continuous variables. *P*-values< 0.05 are considered statistically significant.

Appendix D

Case Report Form (20170919_Version 2.)

A multi-center, randomized, parallel-group, open-label, non-inferiority study to compare the efficacy of intravenous immunoglobulin alone and intravenous immunoglobulin plus high-dose aspirin in acute stage of Kawasaki disease for preventing coronary artery lesion (KIAA trial)
-The second and third years continue study.

Principal Investigator: Ho-Chang Kuo, MD, PhD

Subject Initials: ___ ___ ___

Subject identifier: □□-□□□-□□□

(Center number-screening number-randomization number)

Center number:

01: Kaohsiung Chang Gung Memorial Hospital (KCGMH)

02: Linkou Chang Gung Memorial Hospital (LCGMH)

03: National Taiwan University Hospital (NTUH)

04: Mackay Memorial Hospital (MKMH)

05: Taichung Veterans General Hospital (TVGH)

06: China Medical University Hospital (CMUH)

07: Kaohsiung Veterans General Hospital (KVGH)

Screening numbers are assigned at each site sequentially from 001.

Randomization numbers are assigned centralized (KCGMH-CTC) sequentially from 001.

Screen Visit
(Visit 1, Day -3 to 0)

Visit Date: 20□□/□□/□□ (yyyy/mm/dd)

Admission (or emergency room) date: 20□□/□□/□□ (yyyy/mm/dd)

Fever start date: 20□□/□□/□□/□□ (yyyy/mm/dd/hh)

Demographic Data

Gender: □Male □Female, **Birthday:** □□□□/□□/□□ (yyyy/mm/dd)

Body weight:□□.□Kg, **Body height:**□□□.□cm, **Body temperature:**□□.□ ℃

Baseline laboratory data date: 20□□/□□/□□ (yyyy/mm/dd)

Item	Value	Unit	Item	Value	Unit	Item	Value	Unit
WBC		1000/ul	MCHC		gHb/dl	Monocyte		%
RBC		Million/ul	RDW-SD		%	Eosinophil		%
HGB		g/dl	RDW-CV		%	Basophil		%
HCT		%	PLT		1000/ul	AST/GOT		U/L
MCV		fL	Segment		%	ALT/GPT		U/L
MCH		pg/cell	Lymphocyte		%	CRP		mg/L

Screen Visit

(Visit 1, Day -3 to 0)

Inclusion criteria:

☐Y ☐N 1. Male or female, age less than 6 years old

☐Y ☐N 2. Fulfilled the AHA criteria for KD as below:

☐(1) Fever (more than 38.0℃ear temperature) ≧ 5 days, and 4 of the 5 following symptoms
☐(2) Diffuse mucosal inflammation (strawberry tongue, dry and fissured lips)
☐(3) Bilateral non-purulent conjunctivitis
☐(4) Dysmorphous skin rashes
☐(5) Indurative edematous change over the hands and feet, or desquamation over the finger or toe tip
☐(6) Cervical lymphadenopathy (one or more nodule at least 1.5 cm in diameter)

☐Y ☐N 3. Informed consent form (ICF) signed by the patient or a legal guardian

Exclusion criteria

☐Y ☐N 1. Symptoms not full fit the KD criteria
☐Y ☐N 2. Acute fever for ≦ 5 days or >10 days
☐Y ☐N 3. IVIG treatment at other hospital before refers to study site
☐Y ☐N 4. Treatment with corticosteroids, other than inhaled forms, in the previous 2 weeks
☐Y ☐N 5. Presence of a disease known to mimic Kawasaki disease (such as systemic juvenile idiopathic arthritis, Steven-Johnson Syndrome, Macrophage activation syndrome)
☐Y ☐N 6. Previous diagnosis of KD
☐Y ☐N 7. Inability to take aspirin (history of hypersensitivity to aspirin, G6PD deficiency, intolerance to aspirin)
☐Y ☐N 8. Inability to take IVIG (history of allergic reactions for IVIG, allergic reactions when testing dose of IVIG, selective IgA deficiency)
☐Y ☐N 9. Afebrile before enrolment
☐Y ☐N 10. Concomitant severe medical disorders (e.g. immunodeficiency, chromosomal anomalies, congenital heart diseases, metabolic diseases, nephritis, collagen diseases)
☐Y ☐N 11. Suspected severe infectious disease (e.g. sepsis, septic meningitis, peritonitis, bacterial pneumonia, varicella, and influenza)
☐Y ☐N 12. Conditions judged by the investigator as unsuitable for this trial

Is the subject eligible to enter the trial? ☐ YES ☐NO

I have reviewed all data recorded on all pages of this visit and certify that they are accurate, complete and consistent to the subject's record.	**Investigator's Signature**	**Date**

Randomization Visit
(Visit 2, Day 0 to 1)

Randomization number assignment date: 20□□/□□/□□ (yyyy/mm/dd)

Randomization number: □□□

□ **Test group:**

Patient will receive IVIG (2g/kg) in 10-12 hours alone, **without** high-dose aspirin. After fever subsides, low-dose aspirin (3-5mg/kg/day) will be prescribed until 6-8 weeks.

□ **Standard group:**

Patient will receive IVIG (2g/kg) in 10-12 hours plus high-dose aspirin (80-100mg/kg/day, divided into four doses) till fever subside. After fever subsides, low-dose aspirin (3-5mg/kg/day) will be prescribed until 6-8 weeks.

I have reviewed all data recorded on all pages of this visit and certify that they are accurate, complete and consistent to the subject's record.	**Investigator's Signature**	**Date**

Treatment Visit
(Visit 3, Day 1)

IVIG treatment:

start: 20□□/□□/□□/□□, end: 20□□/□□/□□/□□ (yyyy/mm/dd/hh)

High-dose aspirin treatment □None

start: 20□□/□□/□□/□□, end: 20□□/□□/□□/□□ (yyyy/mm/dd/hh)

Low-dose aspirin treatment

start: 20□□/□□/□□/□□, end: 20□□/□□/□□/□□ (yyyy/mm/dd/hh)

Body temperature (q4h)

**Day 1**	℃	**Day 2**	℃	**Day 3**	℃	**Day 4**	℃	**Day 5**	℃
:		:		:		:		:	
:		:		:		:		:	
:		:		:		:		:	
:		:		:		:		:	
:		:		:		:		:	
:		:		:		:		:	

Fever subside at: 20□□/□□/□□/□□ (yyyy/mm/dd/hh)

Antipyretic Drugs: □Ibuprofen □Acetaminophen □Diclofenac

□Others: _______________

IVIG resistance: □YES □No

Treatment for IVIG resistance: □Second dose of IVIG

□Others: ___________________

I have reviewed all data recorded on all pages of this visit and certify that they are accurate, complete and consistent to the subject's record.	**Investigator's Signature**	**Date**

Day 5 follow up Visit

(Visit 4, Day 3 to 7)

Day 5 follow up laboratory data date: 20□□/□□/□□ (yyyy/mm/dd)

Item	Value	Unit	Item	Value	Unit	Item	Value	Unit
WBC		1000/ul	MCHC		gHb/dl	Monocyte		%
RBC		Million/ul	RDW-SD		%	Eosinophil		%
HGB		g/dl	RDW-CV		%	Basophil		%
HCT		%	PLT		1000/ul	AST/GOT		U/L
MCV		fL	Segment		%	ALT/GPT		U/L
MCH		pg/cell	Lymphocyte		%	CRP		mg/L

Day 5 follow up 2D echocardiography date: 20□□/□□/□□ (yyyy/mm/dd)

Performer: ___________________

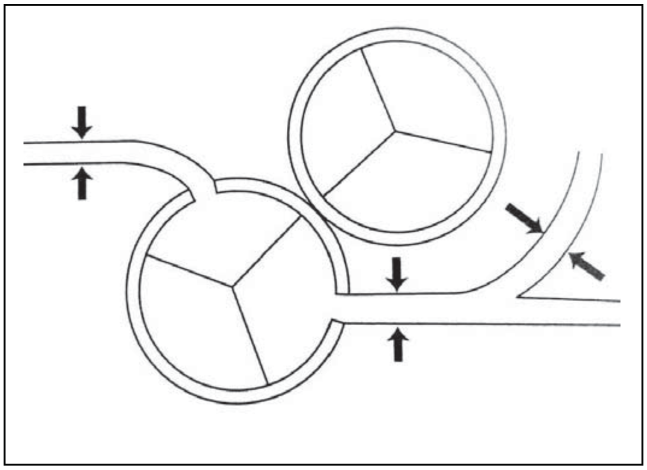

Body weight: □□.□ Kg

Body height: □□□.□ cm

LMCA: _____mm, Z score:_____

LAD: _____mm, Z score:_____

RCA: _____mm, Z score:_____

Coronary artery lesion: □Yes □No

Lesion type: _________________

Discharge date: 20□□/□□/□□ (yyyy/mm/dd)

I have reviewed all data recorded on all pages of this visit and certify that they are accurate, complete and consistent to the subject's record.	**Investigator's Signature**	**Date**

Week 6-8 follow up Visit
(Visit 5, Day 39 to 59)

Week 6-8 follow up laboratory data date: 20□□/□□/□□ (yyyy/mm/dd)

Item	Value	Unit	Item	Value	Unit	Item	Value	Unit
WBC		1000/ul	MCHC		gHb/dl	Monocyte		%
RBC		Million/ul	RDW-SD		%	Eosinophil		%
HGB		g/dl	RDW-CV		%	Basophil		%
HCT		%	PLT		1000/ul	AST/GOT		U/L
MCV		fL	Segment		%	ALT/GPT		U/L
MCH		pg/cell	Lymphocyte		%	CRP		mg/L

Week 6-8 follow up 2D echocardiography date: 20□□/□□/□□ (yyyy/mm/dd)

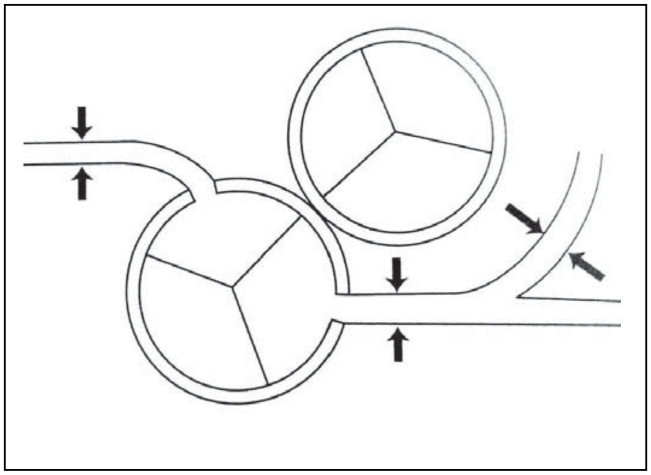

Performer: ___________________

Body weight: □□.□ Kg

Body height: □□□.□ cm

LMCA: _____mm, Z score:_____

LAD: _____mm, Z score:_____

RCA: _____mm, Z score:_____

Coronary artery lesion: □Yes □No

Lesion type: _________________

I have reviewed all data recorded on all pages of this visit and certify that they are accurate, complete and consistent to the subject's record.	Investigator's Signature	Date

Month 6 follow up Visit

(Visit 6, Day 150 to 210)

Month 6 follow up 2D echocardiography date: 20☐☐/☐☐/☐☐ (yyyy/mm/dd)

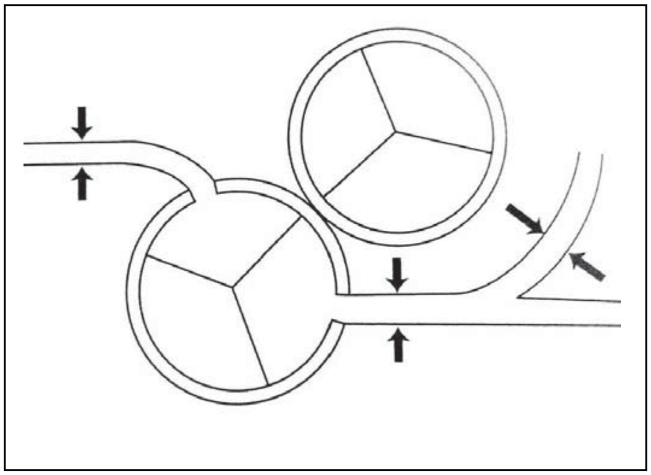

Performer: ___________________

Body weight: ☐☐.☐ Kg

Body height: ☐☐☐.☐ cm

LMCA: _____mm, Z score:_____

LAD: _____mm, Z score:_____

RCA: _____mm, Z score:_____

Coronary artery lesion: ☐Yes ☐No

Lesion type: _________________

Exit Form

Exit date: 20☐☐/☐☐/☐☐

Complete study: ☐YES ☐No

Withdraw: ☐YES (if YES, choose the cause) ☐No

☐ 1. Participant or his/her legal guardian decides to withdraw his/her ICF.

☐ 2. Participant has allergic reaction during IVIG or aspirin treatment.

☐ 3. Participant develops AE or SAE that the investigator considered a cessation of the study treatment is necessary.

☐ 4. Participant is lost of follow up.

☐ 5. Investigator considers that the participant is no longer physically and/or psychologically feasible to remain in the study.

I have reviewed all data recorded on all pages of this visit and certify that they are accurate, complete and consistent to the subject's record.	**Investigator's Signature**	**Date**

Medical history:

☐ None

☐ YES. If YES, describe condition

Diagnosis:____

(Start date: ☐☐☐☐/☐☐/☐☐, Still present ☐Yes ☐No, End date: ☐☐☐☐/☐☐/☐☐)

Diagnosis:____

(Start date: ☐☐☐☐/☐☐/☐☐, Still present ☐Yes ☐No, End date: ☐☐☐☐/☐☐/☐☐)

Previous and Concomitant medications: ☐None

Name	Dose	Unit	Route	Frequency	Start date(YYYY/mm/dd)<br>End date(YYYY/mm/dd)
					20☐☐/☐☐/☐☐<br>20☐☐/☐☐/☐☐
					20☐☐/☐☐/☐☐<br>20☐☐/☐☐/☐☐
					20☐☐/☐☐/☐☐<br>20☐☐/☐☐/☐☐
					20☐☐/☐☐/☐☐<br>20☐☐/☐☐/☐☐

I have reviewed all data recorded on all pages of this visit and certify that they are accurate, complete and consistent to the subject's record.	**Investigator's Signature**	**Date**

Adverse Event: ☐None

Event (one record per event)	Start: mm/dd/hh end: mm/dd/hh	Intensity	Treatment/Comment
**1.**			
**2.**			
**3.**			
**4.**			
**5.**			
**6.**			
**7.**			
**8.**			

I have reviewed all data recorded on all pages of this visit and certify that they are accurate, complete and consistent to the subject's record.	**Investigator's Signature**	**Date**
