# Supplementary material for: Intravenous Immunoglobulin Alone for Coronary Artery Lesion Treatment of Kawasaki Disease: A Randomized Clinical Trial
Source: JAMA Netw Open. 2025 Apr 3;8(4):e253063. doi: 10.1001/jamanetworkopen.2025.3063 (PMC11969286; doi:10.1001/jamanetworkopen.2025.3063)
Supplement: Supplement 2. — eMethods eAppendix 1. Clinical Trial Design and Baseline Characteristics eAppendix 2. Categorized Artery and KD Subgroup Analysis eTable 1. Baseline Characteristics and Group Distribution of Participants in the Study eTable 2. Analysis of Variance of the Clinical Parameters in Terms of the Group, Time, and Their Interaction eTable 3. Comparison of IVIG Treatment Resistance eTable 4. Prophylactic Effect: Comparison Frequency of Abnormal Coronary Arteries Diameter in Term of Groups Type and Time eTable 5. Comparison Frequency of Abnormal Coronary Arteries Diameter in Liver, Band, Node, and Young Age Clusters eTable 6. Comparison Frequency of Abnormal Coronary Arteries Diameter-Liver Cluster eTable 7. Comparison Frequency of Abnormal Coronary Arteries Diameter-Band Cluster eTable 8. Comparison Frequency of Abnormal Coronary Arteries Diameter-Node Cluster eTable 9. Comparison Frequency of Abnormal Coronary Arteries Diameter-Young Age Onset Cluster eTable 10. Comparison Frequency of Abnormal Coronary Arteries Diameter-Subgroup Populations From Liver, Band, Node, and Young Age Clusters eTable 11. Comparison Frequency of Abnormal Coronary Arteries Diameter-Low CRP Subgroup (CRP ≤ 100 mg/L) eTable 12. Comparison Frequency of Abnormal Coronary Arteries Diameter-High CRP Subgroup (CRP > 100 mg/L) eTable 13. Comparison Frequency of Abnormal Coronary Arteries Diameter-Low Platelet Count Subgroup (Platelet < 400 × 103/μL) eTable 14. Comparison Frequency of Abnormal Coronary Arteries Diameter-High Platelet Count Subgroup (Platelet ≥ 400 × 103/μL) eReferences [file jamanetwopen-e253063-s002.pdf]

Supplemental Online Content

Kuo HC, Lin MC, Kao CC, et al. Intravenous immunoglobulin alone for coronary artery lesion treatment of Kawasaki disease: a randomized clinical trial. *JAMA Netw Open*. 2025;8(4):e253063. doi:10.1001/jamanetworkopen.2025.3063

eMethods
eAppendix 1. Clinical Trial Design and Baseline Characteristics
eAppendix 2. Categorized Artery and KD Subgroup Analysis
eTable 1. Baseline Characteristics and Group Distribution of Participants in the Study
eTable 2. Analysis of Variance of the Clinical Parameters in Terms of the Group, Time, and Their Interaction
eTable 3. Comparison of IVIG Treatment Resistance
eTable 4. Prophylactic Effect: Comparison Frequency of Abnormal Coronary Arteries Diameter in Term of Groups Type and Time
eTable 5. Comparison Frequency of Abnormal Coronary Arteries Diameter in Liver, Band, Node, and Young Age Clusters
eTable 6. Comparison Frequency of Abnormal Coronary Arteries Diameter-Liver Cluster
eTable 7. Comparison Frequency of Abnormal Coronary Arteries Diameter-Band Cluster
eTable 8. Comparison Frequency of Abnormal Coronary Arteries Diameter-Node Cluster
eTable 9. Comparison Frequency of Abnormal Coronary Arteries Diameter-Young Age Onset Cluster
eTable 10. Comparison Frequency of Abnormal Coronary Arteries Diameter-Subgroup Populations From Liver, Band, Node, and Young Age Clusters
eTable 11. Comparison Frequency of Abnormal Coronary Arteries Diameter-Low CRP Subgroup (CRP ≤ 100 mg/L)
eTable 12. Comparison Frequency of Abnormal Coronary Arteries Diameter-High CRP Subgroup (CRP > 100 mg/L)
eTable 13. Comparison Frequency of Abnormal Coronary Arteries Diameter-Low Platelet Count Subgroup (Platelet < 400 × 10^3/μL)
eTable 14. Comparison Frequency of Abnormal Coronary Arteries Diameter-High Platelet Count Subgroup (Platelet ≥ 400 × 10^3/μL)
eReferences

This supplemental material has been provided by the authors to give readers additional information about their work.

© 2025 Kuo HC et al. *JAMA Network Open*.

eMethods

Enrolled medical centers:

The clinical trial was conducted at five medical centers in Taiwan, namely Linkou Chang Gung Memorial Hospital, Kaohsiung Chang Gung Memorial Hospital, Taichung Veterans General Hospital, Kaohsiung Veterans General Hospital, and Tungs' Taichung Metro Harbor Hospital.

Participants:

Individuals under the age of 6 meeting the criteria for KD set by the American Heart Association (AHA) were eligible for enrollment in this study.

Inclusion criteria:

1. Male or female, under the age of 6 years old. 2. Fulfilled the AHA criteria for KD as explained: (1) Fever (more than 38.0 °C ear temperature) ≥ 5 days, as well as 4 of the 5 following symptoms; (2) Diffuse mucosal inflammation (strawberry tongue, dry and cracked lips); (3) Bilateral non-purulent conjunctivitis; (4) Dysmorphous skin rashes; (5) Indurative edematous change over the hands and feet, or desquamation over the fingertips or toes; (6) Cervical lymphadenopathy (one or more nodule at least 1.5 cm in diameter). 3. An informed consent form signed by the patient or a legal guardian.

Exclusion criteria:

1. Had symptoms that did not completely match the KD criteria. 2. Had an acute fever for < 5 days and > 10 days. 3. IVIG treatment at another hospital before being referred to the study center. 4. Treatment with corticosteroids, other than the inhaled form, in the two weeks prior to joining the study. 5. The presence of a disease known to mimic Kawasaki disease (such as adenovirus infection, toxic shock syndrome etc.). 6. Previous KD diagnosis. 7. Inability to take aspirin (such as history of hypersensitivity to aspirin, G6PD deficiency, recent herpes zoster infection or vaccination, etc.). 8. A febrile prior to enrollment. 9. Severe concomitant medical disorders (e.g., immunodeficiency, congenital heart diseases, chromosomal anomalies, metabolic diseases, collagen diseases, nephritis, etc.). 10. Suspected to have an infectious disease, including sepsis, septic meningitis, peritonitis, bacterial pneumonia, varicella, and influenza. 11. Judged by the researcher to be unsuitable for this trial.

Withdrawal criteria:

1. The participant or his/her legal guardian decides to withdraw his/her informed consent. 2. The participant is lost for follow-up. 3. The researcher considers the participant to no longer be physically and/or psychologically fit to remain in the study. 4. The participant develops an adverse event (AE) such that the researcher considers stopping the study treatment necessary.

All participants completed a structured questionnaire to gather demographic data such as age, gender, and ethnicity.

Outcome and variables assessment:

© 2025 Kuo HC et al. *JAMA Network Open*.

The commonly accepted understanding of coronary artery lesions (CAL), also known as coronary artery abnormalities (CAA), is based on diagnostic criteria set by the Japanese Ministry of Health. These criteria involve a maximum internal diameter exceeding 3 mm in children under 5 years old or exceeding 4 mm in children aged 5 years and above. Furthermore, the criteria encompass the presence of luminal irregularity, a segmental lumen 1.5 times larger than an adjacent one, or a visibly irregular luminal contour with a Z score exceeding 2.5 standard deviations[1-7].

We monitored the body temperature of participants at six-hour intervals. CAL were defined as a luminal diameter exceeding 3.0 mm in children under the age of five or exceeding 4.0 mm in those aged five or older, when the internal diameter of a segment was 1.5 times or greater than that of an adjacent segment, or when the luminal contour was distinctly irregular, with a Z-score of >2.5 standard deviations. The body weight and height used for calculating the Z-score were obtained from the Taiwan Society of Pediatric Cardiology website (http://www.tspc.org.tw/service/z-score.asp). Absolute diameter measurements of coronary artery can be used to evaluate the patients CAL during the clinical trial timeframe. Adjusted Z scores integrated height, weight, age and gender demographic metrics for the normalized evaluation of each patients' coronary diameters change. Therefore, two groups with variable demographic metrics can be compared. The primary endpoint was identified as the formation of CAL at 6-8 weeks after enrollment. Pediatric cardiologists, unaware of the assigned treatment group, determined the primary endpoint of CAL. The secondary objective was to compare the rate of IVIG resistance, defined as persistent or recrudescent fever for at least 48 hours but no more than seven days after completing the initial IVIG treatment. Additionally, the duration of fever after IVIG treatment, length of hospitalization, liver enzyme levels, gastrointestinal symptoms, complete blood count (CBC)/white blood count (WBC), and C-reactive protein (CRP) between the groups were also recorded for analysis. Adverse events (AE) and serious adverse events (SAE) were recorded, and the severity of the AE was assessed based on the Common Terminology Criteria for Adverse Events (CTCAE) version 4.03, following our established publication protocol[8].

Sample size:

The sample size determination is based on the results of the retrospective data. The estimated proportions of CAL are 17% after IVIG treatment combined with high-dose aspirin and 15.4% after IVIG alone. We consider a difference less than 10% as having no clinical significance. The following contents are the statistical basis for estimating sample size in this trial:

H_0: $P_T - P_S \leqq \delta$

H_A: $P_T - P_S > \delta$

P_T: CAL-free rate in IVIG alone group=0.846.

P_S: CAL-free rate in IVIG+ASA group=0.83.

$\delta = -0.1$.

© 2025 Kuo HC et al. *JAMA Network Open*.

$$n = (Z\alpha + Z\beta)^2/(\varepsilon-\delta)^2\ [P_T(1-P_T) + P_S(1-P_S)]$$

Assuming that the type I and type II error rates are $\alpha = 0.05$ and $\beta = 0.2$, respectively, the clinically meaningful difference δ is − 0.1. The CAL-free rate of the test group (IVIG alone) P_T is 0.846 while that of the standard group (IVIG + ASA) P_S is 0.83. Define $\varepsilon = P_T - P_S$.

Allocation and randomization:

The eligible participants will be randomized to either the IVIG treatment alone or the IVIG plus high-dose aspirin treatment at an equal ratio. The researcher or his/her delegate will contact the Clinical Trial Center of Kaohsiung Chang Gung Memorial Hospital (KCGMH- CTC) once the patients have been confirmed to fulfill all the inclusion/ exclusion criteria. The CTC-KCGMH will assign a treatment arm to the patient. Randomization will be categorized by participating institutions using permuted blocks of random sizes, which are not to be disclosed, thus ensuring concealment.

Blinding process:

Both participants and the study's researchers are unblinded to the IVIG alone group or the IVIG plus high-dose aspirin group. The primary endpoint of CAL will be determined by two pediatric cardiologists blinded to the assigned treatment group.

Non-inferiority Comparison:

The distribution of patient characteristics will be summarized and compared between patients randomized to test and standard care groups. The CAL rate will be estimated in IVIG+ASA and IVIG alone group separately. The one-sided 97.5% confidence for the difference in CAL rate will be constructed. If the upper end of the confidence interval for the difference between the IVIG alone group and IVIG+ASA groups was less than 10% the selected non-inferiority margin specified a priori then the non-inferiority is established. One-sided CI for the difference was contained by the range bounded by the prespecified non-inferiority margin. Here, we don't concern p-value directly. From the perspective of hypothesis testing, one can confirm the non-inferiority by testing the null hypothesis that the difference in CAL rate is greater than 10%. In the current setting, the one-sided p-value would be 0.012, suggesting that we should reject this null hypothesis and conclude that the difference in CAL rate is less than 10%. This method is equivalent to the approach based on one-sided CI, which is more transparent in our opinion. For this reason, we choose to establish the non-inferiority based on confidence interval rather than P-value.

© 2025 Kuo HC et al. *JAMA Network Open*.

eAppendix 1. Clinical Trial Design and Baseline Characteristics

1.1 Patient enrollment and experimental design for the clinical trial
The schematic trial design is presented in Figure 2. The overall comparison between the IVIG alone and IVIG+ASA groups is illustrated in Figure 2A: 69 patients were allocated to the IVIG+ASA group, receiving the standard treatment of intravenous immunoglobulin (2 g/kg) along with aspirin (80-100 mg/kg/day) until their fever subsided for 48 hours. The remaining 65 patients were assigned to the IVIG alone group, receiving intravenous immunoglobulin (2 g/kg) alone. Majority of the fever was resolved within 24 hours between the two groups after IVIG treatment. Following the initial treatment, both groups received a daily aspirin dose (3-5 mg/kg) for six weeks. The primary endpoint of this trial is the development of CAL. Patients underwent 2D echocardiography at baseline, weeks 1, 2, 4, and 6-8, and again at 6 months. At 6 weeks and 6 months, the internal diameter and Z-score of the left and right main coronary arteries were calculated. CAL was defined for each age group: over 3.0 mm diameter for children under 5 years, over 4.0 mm for those 5 years or older, or any irregular luminal contour or Z-score exceeding 2.5. Throughout the study period, we also compared the treatment effect and prevention effect, as shown in Figure 2B and 2C, respectively. For the treatment effect, we followed CAL patients (Z score >2.5) at the acute phase to the endpoint. For the prevention effect, we followed non-CAL patients (Z score ≤2.5) at the acute phase to the endpoint.

1.2 Baseline characteristics and group distribution of participants in the study
As shown in eTable 1, the percentage of males in the IVIG+ASA group (62%, n=43) and the IVIG alone group (60%, n=39) did not exhibit a significant difference (P=0.93). Similarly, no statistically significant distinctions were observed between the groups in terms of mean weight (11.13±3.56 Kg vs 11.14±3.58 Kg, P=0.99) or height (81.97±13.85 cm vs 81.79±12.44 cm, P=0.97). Likewise, the mean ages of the two groups were comparable (1.85±1.43 years vs 1.69±1.17 years, P=0.46). Further analysis revealed no significant differences in baseline characteristics between the IVIG+ASA and IVIG alone groups. Specifically, admission days (4.93±1.86 vs 5.32±1.89 days, P=0.22), white blood cell counts (15.11±6.21 vs 13.23±4.80 x1000/μL, P=0.05), hemoglobin levels (11.13±0.94 vs 11.39±1.07 g/dL, P=0.14), platelet counts (350.70±111.20 vs 319.00±90.09 x1000/μL, P=0.07), GOT levels (90.20±120.80 vs 101.30±166.80 U/L, P=0.67), and GPT levels (140.70±139.60 vs 100.30±114.80 U/L, P=0.84) were all similar between the groups. The IVIG+ASA group exhibited significantly higher C-reactive protein levels at baseline compared to the IVIG alone group (68.09 ± 76.66 vs. 41.86 ± 48.06 mg/L, P=0.02). Baseline coronary artery diameters and Z-scores in both the left main coronary artery (LMCA), right coronary artery (RCA), and left anterior descending artery (LAD) did not show significant differences between the groups (2.23±0.48 vs 2.17±0.39 mm, P=0.38; 1.87±0.44 vs 1.95±0.41 mm, P=0.28; and 1.74±0.44 vs 1.76±0.40 mm, P=0.79 for diameter; 1.26±1.00 vs 1.11±0.96, P=0.38; 1.08±0.90 vs 1.24±0.78, P=0.28, and 0.78±0.77 vs 0.84±0.72, P=0.67 for Z-score).

© 2025 Kuo HC et al. *JAMA Network Open*.

1.3 Clinical parameters changes and dynamics during the study period

Clinical parameters changes throughout the study were described in eTable 2. There was a noticeable decrease in C-reactive protein (CRP) levels. Starting from an average baseline of 55.47 mg/L (±65.60 SD), they decreased to just 1.76 mg/L (±8.34 SD) at 6 weeks. Although statistical analysis initially revealed a significant difference between the IVIG+ASA and IVIG alone groups at baseline, this distinction disappeared by the 6-week mark (1.82±8.78 vs 1.70±7.91 mg/L, P=0.94). Overall, CRP levels exhibited a significant decline across the study duration (55.47±65.60 vs 1.76±8.34 mg/L, P<0.001), and interestingly, the specific pattern of this decline showed slight differences between the two groups (68.09±76.66 reduced to 1.82±8.78 vs 41.86±48.06 reduced to 1.70±7.91 mg/L, P=0.03 for interaction effect). Both groups demonstrated a general increase in the average platelet count after IVIG treatment compared to baseline levels. While the average platelet count changed significantly over time (335.20±102.30 vs 376.50±102.10 X1000/ µL, P=0.001), there were no noticeable differences between the two groups at either baseline (350.70±111.20 vs 319.00±90.09 X1000/ µL, P=0.36) or 6 weeks after treatment (372.70±114.20 vs 380.80±87.61 X1000/ µL, P=0.67), and no interaction effect was observed between time and group type (350.70±111.20 increased to 372.70±114.20 vs 319.00±90.09 increased to 380.80±87.61 X1000/ µL, P=0.12). While white blood cell (WBC) levels exhibited a significant difference between the IVIG+ASA and IVIG alone groups at baseline (15.11±6.21 vs 13.23±4.80 X1000/ µL, P=0.04), this distinction disappeared by the 6th week (9.15±3.23 vs 8.64±2.54 X1000/ µL, P=0.34). Overall, WBC levels displayed a significant decrease across the study duration (14.19±5.63 vs 8.90±2.92 X1000/ µL, P<0.001), but the rate of decrease did not show a significant difference between the two groups (15.11±6.21 reduced to 9.15±3.23 vs 13.23±4.80 reduced to 8.64±2.54 X1000/ µL, P=0.23). Hemoglobin levels increased significantly at 6 weeks in both groups (11.26±1.01 vs 12.01±1.02 g/dL, P<0.001). Although baseline levels differed significantly between the groups (11.13±0.94 vs 11.39±1.07 g/dL, P=0.02), this difference vanished by the 6th week (11.86±1.03 vs 12.16±0.98 g/dL, P=0.10). The rate of increase did not exhibit a significant difference between the groups (11.13±0.94 increased to 11.86±1.03 vs 11.39±1.07 increased to 12.16±0.98 g/dL, P=0.87). Two liver enzymes, glutamic oxaloacetic transaminase (GOT) and glutamic pyruvic transaminase (GPT), were monitored over time. Both enzymes showed a significant decrease throughout the study (95.38±143.70 to 39.09±9.29 U/L and 102.50±127.70 to 18.97±8.46 U/L, P<0.001). Interestingly, their levels at baseline and at 6 weeks did not display a significant difference between the two groups (90.20±120.80 vs 101.30±166.80 U/L, P=0.69 for GOT and 104.70±139.60 vs 100.30±114.80 U/L, P=0.87 for GPT), and the rate of decrease over time was also similar (90.20±120.80 reduced to 38.93±10.37 vs 101.30±166.80 reduced to 39.27±7.98 U/L, P=0.70 for GOT and 104.70±139.60 reduced to 18.78±9.51 vs 100.30±114.80 reduced to 19.19±7.21 U/L, P=0.84 for GPT).

© 2025 Kuo HC et al. *JAMA Network Open*.

eAppendix 2. Categorized Artery and KD Subgroup Analysis

2.1 LMCA, LAD and RCA artery Changes and Frequency during study period

As depicted in Table 1, at the commencement of the study, 6.2% of patients in the IVIG alone group and 8.7% in the IVIG+ASA group displayed CAL of the LMCA. At 6 months, these percentages decreased to 1.5% and 0% for the IVIG alone and IVIG+ASA groups, respectively. There was no significant difference observed between the groups in terms of the decreasing trend in the frequency of CAL (P=0.42). The frequency of LMCA CAL exhibited a significant reduction at both 6 weeks (P=0.02) and the 6 months (P=0.006) compared to the baseline.

Following the criteria for CAL with a Z score surpassing 2.5, no patients with CAL in the LAD were detected at any of the three measurement time points in both groups. Therefore, no discernible trend in CAL reduction could be calculated.

Additionally, at baseline, 6.2% of patients in the IVIG alone group and 4.3% in the IVIG+ASA group displayed abnormal RCA diameter. Nevertheless, these percentages decreased to 1.5% and 1.4% at 6 months, with no significant difference observed between the groups (P=0.42). While the RCA CAL exhibited a noticeable decreasing trend at both 6 weeks (P=0.09) and 6 months (P=0.09) compared to the baseline, statistical significance was not achieved.

2.2 Treatment and Prophylactic Effect on LMCA, LAD and RCA Artery Changes and Frequency during Study Period between Two Groups

As depicted in Table 2, at the initiation of the study, 6.2% of patients in the IVIG alone group and 8.7% in the IVIG+ASA group displayed CAL of the LMCA. At 6 months, all CAL patients returned to a normal state in both groups. No significant difference was noted between the groups concerning the decreasing trend in the frequency of CAL (P=0.23). The frequency of LMCA CAL exhibited a significant reduction at both 6 weeks (P=0.006) and 6 months (P=0.001) compared to the baseline.

Following the criteria for CAL with a Z score exceeding 2.5, no patients with CAL in the LAD were detected at any of the three measurement time points in both groups. Therefore, no discernible trend in CAL reduction could be calculated.

Furthermore, at the baseline, 6.2% of patients in the test group and 4.3% in the standard group displayed abnormal RCA diameter. All CAL patients returned to normal in both groups at 6 months, with no significant difference between the groups (P=0.18). The frequency of RCA CAL exhibited a noticeable reduction trend at both 6 weeks (P=0.07) and 6 months (P=0.008) compared to the baseline.

Following treatment effect, we investigated the prophylactic impact on the frequency of CAL over the study duration for patients who developed CAL later after IVIG alone or

© 2025 Kuo HC et al. *JAMA Network Open*.

IVIG+ASA aspirin treatment. We performed the same statistical analysis for the comparison of CAL frequencies, as illustrated in eTable 4. No statistical difference was identified between the IVIG+ASA and IVIG alone groups. However, a minimal number of newly developed CAL cases were observed after treatment: 2 in the IVIG alone group (1 CAL of LMCA and 1 CAL of RCA) and 1 (CAL of RCA) in the IVIG+ASA group.

2.3 Four KD Cluster Subgroups Analysis of Coronary Artery Lesions frequency during study period

A recent exploratory study, examining the heterogeneity of Kawasaki disease, identified four distinct patient clusters based on subjective clinical features and laboratory results. These clusters were categorized as the liver subgroup, band subgroup, node subgroup, and young subgroup. Importantly, these subgroups displayed variations in treatment response and disease outcomes, including the risk of coronary artery aneurysm and the rate of intravenous immunoglobulin resistance. To assess the impact of high-dose aspirin treatment on the development of CAA within these diverse clusters, we further classified patients into four clusters in both the test and standard groups. As presented in eTable 5, we conducted a comparative analysis of the overall CAL rates at baseline, 6 weeks, and 6 months across the four identified clusters. Noticeably, discernible trends of reduced CAL rates were evident in all four clusters at both 6 weeks and 6 months post-treatment compared to the baseline. However, no significant differences between the two groups were observed within the liver, node, or young clusters during the study periods (P=0.42, P>0.99, and P=0.18, respectively). Although a significant difference was noted between the groups in the band cluster based on the P value, it may be inconclusive due to the limited number of cases at baseline within this cluster. A more detailed analysis of CAL rates within each cluster for LMCA, LAD, and RCA was shown in eTables 6 to 9.

As certain criteria for the identified clusters are not mutually exclusive, and some patients may exhibit clinical features aligning with more than one cluster, we conducted further examination of the CAL rates in subgroups of patients with multiple cluster labels. As shown in eTable 10, no statistically significant differences were identified between the standard and test groups in terms of reducing the frequency of CAL across all subgroups (P>0.05).

2.4 Inflammation biomarker-based subgroup analysis of Coronary Artery Changes and Lesions frequency during study period

Kawasaki Disease represents a complex pathological condition characterized by diverse clinical manifestations and outcomes. To gain a deeper understanding of the trial data, we examined the frequency of CAL outcomes across the study period within subgroups defined by inflammation biomarkers, specifically C-reactive protein (CRP) and platelet count. Employing criteria from Benioff Children’s Hospital of the University of California at San Francisco (https://www.ucsfbenioffchildrens.org/medical-tests), we categorized KD patients into CRP high (abnormal range) and low (normal range) subgroups, as well as platelet count high and low subgroups, in each study arm.

© 2025 Kuo HC et al. *JAMA Network Open*.

As shown in Supplementary eTable 11, within the CRP low group (CRP≤100 mg/L), 10.9% and 12.0% of patients in the IVIG alone and IVIG+ASA groups, respectively, exhibited abnormal coronary artery diameter (Z score >2.5) in at least one of the three coronary arteries at baseline. These percentages decreased to 1.8% and 2.0% at 6 months with no significant differences between the groups in terms of the frequency of abnormal CAA throughout the study period (P>0.99). Notably, the frequency of CAL demonstrated a significant decrease from the 6th week (P=0.006) through the end of the study period (6th month P=0.006) compared to the baseline.

For each type of coronary artery, approximately 5.5% of patients in the IVIG alone group and 8.0% in the IVIG+ASA group exhibited CAL in the LMCA at the study's baseline. After 6 months, these percentages dropped to 0% in both groups. No significant difference was found between the groups regarding the decreasing trend in CAL frequency (P=0.18). Notably, the frequency of LMCA CAL demonstrated a significant reduction at both 6 weeks (P=0.03) and 6 months (P=0.007) compared to the baseline. Using the criteria for CAL (Z score >2.5), no patients with CAL in the LAD were identified at any of the three measurement timepoints in both groups, leading to the absence of a calculated CAL reduction trend. Furthermore, at the baseline, 7.3% and 4.0% of patients in the IVIG alone and IVIG+ASA groups, respectively, had abnormal RCA diameter. However, these percentages decreased to 1.8% and 2.0% at 6 months with no significant difference between the groups (P=0.23). Although the RCA CAL exhibited a clear decreasing trend at 6 weeks (P=0.06) and 6 months (P=0.15) compared to the baseline, it did not reach statistical significance.

Subsequently, we assessed the frequency of CAL over the study period for the CRP high group (CRP>100 mg/L) and performed the same statistical analysis for the comparison of CAL frequencies as depicted in Supplementary eTable 12. No statistical difference was observed in this CRP high group between the IVIG+ASA and IVIG alone groups. However, a minimal number of CAL cases were noted (1 in the IVIG alone group and 3 in the IVIG+ASA group at baseline) within this subgroup.

Similarly, based on the platelet count values, we stratified the patients into two subgroups. As illustrated in eTable 13, within the platelet count low group (platelet <400x10^3/uL), a total of 11.1% and 17.4% of patients in the IVIG alone and IVIG+ASA groups, respectively, displayed abnormal coronary artery diameter (Z score >2.5) in at least one of the three coronary arteries at baseline. By the 6th month, these percentages decreased to 3.7% and 0%, respectively. No significant differences were observed between the groups in terms of the frequency of abnormal CAA throughout the study period (P=0.81). Notably, the frequency of CAL exhibited a significant decrease compared to the baseline starting from the 6th week (P=0.005) through the end of the study period (6th month P=0.002).

For each type of coronary artery, approximately 7.4% of patients in the IVIG alone group and 10.9% in the IVIG+ASA group presented with CAL in the LMCA at the study's baseline. At 6 months, these percentages decreased to 1.9% and 0%, respectively. No significant difference was observed between the groups regarding the decreasing trend in the frequency of CAL (P=0.53). Notably, the frequency of LMCA CAL demonstrated a

© 2025 Kuo HC et al. *JAMA Network Open*.

significant reduction at both 6 weeks (P=0.03) and 6 months (P=0.009) of the disease period compared to the baseline. Using the criteria for CAL (Z score >2.5), no patients with CAL in the LAD were identified at any of the three measurement timepoints in both groups, leading to the absence of a calculated CAL reduction trend. Furthermore, at the baseline, 5.6% and 6.5% of patients in the IVIG alone and IVIG+ASA groups, respectively, exhibited abnormal RCA diameter. However, these percentages decreased to 1.9% and 0% at 6 months with no significant difference between the groups (P=0.42). Although the RCA CAL exhibited a clear decreasing trend at 6 weeks (P=0.15) and 6 months (P=0.05) compared to the baseline, it did not reach statistical significance.

We analyzed the occurrence of CAL throughout the study period for the platelet count high group (platelet≥400x10^3/uL) and conducted a similar statistical analysis to compare CAL frequencies, as presented in eTable 14. No statistically significant difference was observed in this platelet high group between the IVIG alone and IVIG+ASA groups. However, only a minimal number of CAL cases were noted (1 in the test group and 1 in the standard group at baseline) within this subgroup.

© 2025 Kuo HC et al. *JAMA Network Open*.

eTable 1. Baseline Characteristics and Group Distribution of Participants in the Study

Variables	Mean ± SD		
	**Total (n=134)**	**IVIG+ASA Group (n=69)**	**IVIG alone Group (n=65)**
Gender			
Male, n (%)	82 (61.2)	43 (62.3)	39 (60. 0)
Female, n (%)	52 (38.8)	26 (37.7)	26 (40.0)
Age, years	1.77±1.31	1.85±1.43	1.69±1.17
Weight, Kg	11.14±3.56	11.13±3.56	11.14±3.58
Height, cm	81.83±13.13	81.87±13.85	81.79±12.44
Admission, days	5.12±1.88	4.93±1.86	5.32±1.89
WBC, x1000/μL	14.19±5.63	15.11±6.21	13.23±4.80
Hemoglobin, g/dL	11.26±1.01	11.13±0.94	11.39±1.07
Platelet, x1000/μL	335.20±102.30	350.70±111.20	319.00±90.09
CRP, mg/L	55.47±65.60	68.09±76.66	41.86±48.06
GOT, U/L	95.38±143.70	90.20±120.80	101.30±166.80
GPT, U/L	102.50±127.70	140.70±139.60	100.30±114.80
LCMA diameter, mm	2.20±0.44	2.23±0.48	2.17±0.39
LCMA Z score	1.19±0.98	1.26±1.00	1.11±0.96
LAD diameter, mm	1.75±0.42	1.74±0.44	1.76±0.40
LAD Z score	0.81±0.74	0.78±0.77	0.84±0.72
RAC diameter, mm	1.91±0.43	1.87±0.44	1.95±0.41
RAC Z score	1.16±0.84	1.08±0.90	1.24±0.78

SD: Standard deviation; WBC: White blood cell count; CRP: C-reactive protein; GOT: Glutamic oxaloacetic transaminase; GPT: Glutamic pyruvic transaminase; LMCA: Left main coronary artery; RCA: Right coronary artery; LAD: Left anterior descending.

© 2025 Kuo HC et al. *JAMA Network Open*.

eTable 2. Analysis of Variance of the Clinical Parameters in Terms of the Group, Time, and Their Interaction

Variable	Time	Mean ± SD			P[a]	P[b]	P[c]
		IVIG+ASA Group	IVIG alone Group	Total			
CRP	Baseline	68.09±76.66	41.86±48.06	55.47±65.60	0.03	<0.001	0.03
	after 6 weeks	1.82±8.78	1.70±7.91	1.76±8.34	0.94		
	after 6 months	N.D.	N.D.	N.D.			
Platelet	Baseline	350.70±111.20	319.00±90.09	335.20±102.30	0.36	0.001	0.12
	after 6 weeks	372.70±114.20	380.80±87.61	376.50±102.10	0.67		
	after 6 months	N.D.	N.D.	N.D.			
WBC	Baseline	15.11±6.21	13.23±4.80	14.19±5.63	0.04	<0.001	0.23
	after 6 weeks	9.15±3.23	8.64±2.54	8.90±2.92	0.34		
	after 6 months	N.D.	N.D.	N.D.			
Hemoglobin	Baseline	11.13±0.94	11.39±1.07	11.26±1.01	0.03	<0.001	0.87
	after 6 weeks	11.86±1.03	12.16±0.98	12.01±1.02	0.10		
	after 6 months	N.D.	N.D.	N.D.			
GOT	Baseline	90.20±120.80	101.30±166.80	95.38±143.70	0.69	<0.001	0.70
	after 6 weeks	38.93±10.37	39.27±7.98	39.09±9.29	0.85		
	after 6 months	N.D.	N.D.	N.D.			
GPT	Baseline	104.70±139.60	100.30±114.80	102.50±127.70	0.87	<0.001	0.84
	after 6 weeks	18.78±9.51	19.19±7.21	18.97±8.46	0.80		
	after 6 months	N.D.	N.D.	N.D.			
LMCA diameter	Baseline	2.23±0.48	2.17±0.39	2.20±0.44	0.90	<0.001	0.17
	after 6 weeks	2.05±0.45	1.99±0.39	2.02±0.42	0.48		
	after 6 months	1.97±0.38	2.07±0.32	2.02±0.35	0.10		
LAD diameter	Baseline	1.74±0.44	1.76±0.40	1.75±0.42	0.19	<0.001	0.68
	after 6 weeks	1.55±0.39	1.59±0.36	1.57±0.38	0.58		
	after 6 months	1.50±0.39	1.61±0.36	1.56±0.38	0.14		
RCA diameter	Baseline	1.87±0.44	1.95±0.41	1.91±0.43	0.16	0.006	0.83
	after 6 weeks	1.75±0.41	1.77±0.39	1.76±0.40	0.75		
	after 6 months	1.78±0.37	1.85±0.34	1.81±0.36	0.29		

© 2025 Kuo HC et al. *JAMA Network Open*.

([a]Difference between two group, [b]Difference between the three times, [c]Interaction effect between time and group)

CRP: C-reactive protein, WBC: White blood cell; GOT: Glutamic oxaloacetic transaminase, GPT: Glutamic pyruvic transaminase, LMCA: Left main coronary artery, RCA: Right coronary artery, LAD: Left anterior descending.

eTable 3. Comparison of IVIG Treatment Resistance

Group	IVIG Resistance	IVIG Responsive	P-value
IVIG alone	3	62	0.94
IVIG+ASA	3	66	

© 2025 Kuo HC et al. *JAMA Network Open*.

eTable 4. Prophylactic Effect: Comparison Frequency of Abnormal Coronary Arteries Diameter in Term of Groups Type and Time

CAL	Group	Abnormal (Z score>2.5)	Baseline	At 6 weeks	At 6 months	P-value[b]
LMCA	IVIG alone	Yes	0 (0.0)	0 (0.0)	1 (1.5)	>0.99
		No	65 (100.0)	65 (100.0)	64 (98.5)	
	IVIG+ASA	Yes	0 (0.0)	1 (1.4)	0 (0.0)	
		No	69 (100.0)	68 (98.5)	69 (100.0)	
	P-value[a]		Ref	0.32	0.32	
LAD	IVIG alone	Yes	0 (0.0)	0 (0.0)	0 (0.0)	N.A.
		No	65 (100.0)	65 (100.0)	65 (100.0)	
	IVIG+ASA	Yes	0 (0.0)	0 (0.0)	0 (0.0)	
		No	69 (100.0)	69 (100.0)	69 (100.0)	
	P-value[a]		Ref	N.A.	N.A.	
RCA	IVIG alone	Yes	0 (0.0)	0 (0.0)	1 (1.5)	0.42
		No	65 (100.0)	65 (100.0)	64 (98.5)	
	IVIG+ASA	Yes	0 (0.0)	1 (1.4)	1 (1.4)	
		No	69 (100.0)	68 (98.5)	68 (98.5)	
	P-value[a]		Ref	0.32	0.16	
Overall	IVIG alone	Yes	0 (0.0)	0 (0.0)	2 (3.1)	>0.99
		No	65 (100.0)	65 (100.0)	63 (96.9)	
	IVIG+ASA	Yes	0 (0.0)	1 (1.4)	1 (1.4)	
		No	69 (100.0)	68 (98.5)	68 (98.5)	
	P-value[a]		Ref	0.32	0.08	

LMCA: Left main coronary artery, RCA: Right coronary artery, LAD: Left anterior descending; [a]CAL frequency difference between the 6th week or the 6th month and baseline; [b]CAL frequency changes between IVIG+ASA and IVIG alone groups during study period.

© 2025 Kuo HC et al. *JAMA Network Open*.

eTable 5. Comparison Frequency of Abnormal Coronary Arteries Diameter in Liver, Band, Node, and Young Age Clusters

Clusters	Group	Abnormal (Z score>2.5)	Baseline	At 6 weeks	At 6 months	P-value[b]
Liver[c]	IVIG alone (N=10)	Yes	2 (20.0)	0 (0.0)	0 (0.0)	0.42
		No	8 (80.0)	10 (100.0)	10 (100.0)	
	IVIG+ASA (N=17)	Yes	2 (11.8)	1 (5.9)	0 (0.0)	
		No	15 (88.2)	16 (94.1)	17 (100.0)	
	P-value[a]		Ref	0.16	0.04	
Band[d]	IVIG alone (N=30)	Yes	2 (6.7)	1 (3.3)	1 (3.3)	<0.001
		No	28 (93.3)	29 (96.7)	29 (96.7)	
	IVIG+ASA (N=15)	Yes	1 (6.7)	0 (0.0)	0 (0.0)	
		No	14 (93.3)	15 (100.0)	15 (100.0)	
	P-value[a]		Ref	0.31	0.31	
Node[e]	IVIG alone (N=27)	Yes	4 (14.8)	1 (3.7)	0 (0.0)	>0.99
		No	23 (85.2)	26 (96.3)	27 (100.0)	
	IVIG+ASA (N=27)	Yes	3 (11.1)	1 (3.7)	1 (3.7)	
		No	24 (88.9)	26 (96.3)	26 (96.3)	
	P-value[a]		Ref	0.08	0.03	
Young[f]	IVIG alone (N=44)	Yes	5 (11.4)	0 (0.0)	1 (2.3)	0.18
		No	39 (88.6)	44 (100.0)	43 (97.7)	
	IVIG+ASA (N=43)	Yes	6 (14.0)	1 (2.3)	1 (2.3)	
		No	37 (86.0)	42 (97.7)	42 (97.7)	
	P-value[a]		Ref	0.003	0.01	

[a]CAL frequency difference between the 6th week or the 6th month and baseline; [b]CAL frequency changes between IVIG+ASA and IVIG alone groups during study period.

[c]GOT>106 U/L or GPT>106 U/L;

[d]Platelet≤308x10^3/uL and WBC≤12.2x10^3/uL;

[e]Platelet>308x10^3/uL and WBC>12.2x10^3/uL;

[f]Age≤1.7 years old

© 2025 Kuo HC et al. *JAMA Network Open*.

eTable 6. Comparison Frequency of Abnormal Coronary Arteries Diameter-Liver Cluster

CAL	Group	Abnormal (Z score>2.5)	Baseline	At 6 weeks	At 6 months	P-value[b]
LMCA	IVIG alone	Yes	2 (20.0)	0 (0.0)	0 (0.0)	>0.99
		No	8 (80.0)	10 (100.0)	10 (100.0)	
	IVIG+ASA	Yes	1 (5.9)	1 (5.9)	0 (0.0)	
		No	16 (94.1)	16 (94.1)	17 (100.0)	
	P-value[a]		Ref	0.30	0.08	
LAD	IVIG alone	Yes	0 (0.0)	0 (0.0)	0 (0.0)	N.A.
		No	10 (100.0)	10 (100.0)	10 (100.0)	
	IVIG+ASA	Yes	0 (0.0)	0 (0.0)	0 (0.0)	
		No	17 (100.0)	17 (100.0)	17 (100.0)	
	P-value[a]		Ref	N.A.	N.A.	
RCA	IVIG alone	Yes	0 (0.0)	0 (0.0)	0 (0.0)	
		No	10 (100.0)	10 (100.0)	10 (100.0)	
	IVIG+ASA	Yes	1 (5.9)	0 (0.0)	0 (0.0)	0.42
		No	16 (94.1)	17 (100.0)	17 (100.0)	
	P-value[a]		Ref	0.31	0.31	
Overall	IVIG alone	Yes	2 (20.0)	0 (0.0)	0 (0.0)	0.42
		No	8 (80.0)	10 (100.0)	10 (100.0)	
	IVIG+ASA	Yes	2 (11.8)	1 (5.9)	0 (0.0)	
		No	15 (88.2)	16 (94.1)	17 (100.0)	
	P-value[a]		Ref	0.16	0.04	

LMCA: Left main coronary artery, RCA: Right coronary artery, LAD: Left anterior descending; [a]CAL frequency difference between the 6th week or the 6th month and baseline; [b]CAL frequency changes between IVIG+ASA and IVIG alone groups during study period.

© 2025 Kuo HC et al. *JAMA Network Open*.

eTable 7. Comparison Frequency of Abnormal Coronary Arteries Diameter-Band Cluster

CAL	Group	Abnormal (Z score>2.5)	Baseline	At 6 weeks	At 6 months	P-value[b]
LMCA	IVIG alone	Yes	1 (3.3)	0 (0.0)	0 (0.0)	0.42
		No	29 (96.7)	30 (100.0)	30 (100.0)	
	IVIG+ASA	Yes	0 (0.0)	0 (0.0)	0 (0.0)	
		No	15 (100.0)	15 (100.0)	15 (100.0)	
	P-value[a]		Ref	0.32	0.32	
LAD	IVIG alone	Yes	0 (0.0)	0 (0.0)	0 (0.0)	N.A.
		No	30 (100.0)	30 (100.0)	30 (100.0)	
	IVIG+ASA	Yes	0 (0.0)	0 (0.0)	0 (0.0)	
		No	15 (100.0)	15 (100.0)	15 (100.0)	
	P-value[a]		Ref	N.A.	N.A.	
RCA	IVIG alone	Yes	2 (6.7)	1 (3.3)	1 (3.3)	<0.001
		No	28 (93.3)	29 (96.7)	29 (96.7)	
	IVIG+ASA	Yes	1 (6.7)	0 (0.0)	0 (0.0)	
		No	14 (93.3)	15 (100.0)	15 (100.0)	
	P-value[a]		Ref	0.31	0.31	
Overall	IVIG alone	Yes	2 (6.7)	1 (3.3)	1 (3.3)	<0.001
		No	28 (93.3)	29 (96.7)	29 (96.7)	
	IVIG+ASA	Yes	1 (6.7)	0 (0.0)	0 (0.0)	
		No	14 (93.3)	15 (100.0)	15 (100.0)	
	P-value[a]		Ref	0.31	0.31	

LMCA: Left main coronary artery, RCA: Right coronary artery, LAD: Left anterior descending; [a]CAL frequency difference between the 6th week or the 6th month and baseline; [b]CAL frequency changes between IVIG+ASA and IVIG alone groups during study period.

© 2025 Kuo HC et al. *JAMA Network Open*.

eTable 8. Comparison Frequency of Abnormal Coronary Arteries Diameter-Node Cluster

CAL	Group	Abnormal (Z score>2.5)	Baseline	At 6 weeks	At 6 months	P-value[b]
LMCA	IVIG alone	Yes	2 (7.4)	0 (0.0)	0 (0.0)	0.67
		No	25 (92.6)	27 (100.0)	27 (100.0)	
	IVIG+ASA	Yes	1 (3.7)	1 (3.7)	1 (3.7)	
		No	26 (96.3)	26 (96.3)	26 (96.3)	
	P-value[a]		Ref	0.31	0.31	
LAD	IVIG alone	Yes	0 (0.0)	0 (0.0)	0 (0.0)	N.A.
		No	27 (100.0)	27 (100.0)	27 (100.0)	
	IVIG+ASA	Yes	0 (0.0)	0 (0.0)	0 (0.0)	
		No	27 (100.0)	27 (100.0)	27 (100.0)	
	P-value[a]		Ref	N.A.	N.A.	
RCA	IVIG alone	Yes	2 (7.4)	1 (3.7)	0 (0.0)	
		No	25 (92.6)	26 (96.3)	27 (100.0)	
	IVIG+ASA	Yes	2 (7.4)	1 (3.7)	0 (0.0)	>0.99
		No	25 (92.6)	26 (96.3)	27 (100.0)	
	P-value[a]		Ref	0.40	0.04	
Overall	IVIG alone	Yes	4 (14.8)	1 (3.7)	0 (0.0)	>0.99
		No	23 (85.2)	26 (96.3)	27 (100.0)	
	IVIG+ASA	Yes	3 (11.1)	1 (3.7)	1 (3.7)	
		No	24 (88.9)	26 (96.3)	26 (96.3)	
	P-value[a]		Ref	0.08	0.03	

LMCA: Left main coronary artery, RCA: Right coronary artery, LAD: Left anterior descending; [a]CAL frequency difference between the 6th week or the 6th month and baseline; [b]CAL frequency changes between IVIG+ASA and IVIG alone groups during study period.

© 2025 Kuo HC et al. *JAMA Network Open*.

eTable 9. Comparison Frequency of Abnormal Coronary Arteries Diameter-Young Age Onset Cluster

CAL	Group	Abnormal (Z score>2.5)	Baseline	At 6 weeks	At 6 months	P-value[b]
LMCA	IVIG alone	Yes	4 (9.1)	0 (0.0)	1 (2.3)	>0.99
		No	40 (90.9)	44 (100.0)	43 (97.7)	
	IVIG+ASA	Yes	4 (9.3)	1 (2.3)	0 (0.0)	
		No	39 (90.7)	42 (97.7)	43 (100.0)	
	P-value[a]		Ref	0.02	0.02	
LAD	IVIG alone	Yes	0 (0.0)	0 (0.0)	0 (0.0)	N.A.
		No	44 (100.0)	44 (100.0)	44 (100.0)	
	IVIG+ASA	Yes	0 (0.0)	0 (0.0)	0 (0.0)	
		No	43 (100.0)	43 (100.0)	43 (100.0)	
	P-value[a]		Ref	N.A.	N.A.	
RCA	IVIG alone	Yes	2 (4.5)	0 (0.0)	0 (0.0)	0.18
		No	42 (95.5)	44 (100.0)	44 (100.0)	
	IVIG+ASA	Yes	2 (4.7)	1 (2.3)	1 (2.3)	
		No	41 (95.3)	42 (97.7)	42 (97.7)	
	P-value[a]		Ref	0.17	0.17	
Overall	IVIG alone	Yes	5 (11.4)	0 (0.0)	1 (2.3)	0.18
		No	39 (88.6)	44 (100.0)	43 (97.7)	
	IVIG+ASA	Yes	6 (14.0)	1 (2.3)	1 (2.3)	
		No	37 (86.0)	42 (97.7)	42 (97.7)	
	P-value[a]		Ref	0.003	0.01	

LMCA: Left main coronary artery, RCA: Right coronary artery, LAD: Left anterior descending; [a]CAL frequency difference between the 6th week or the 6th month and baseline; [b]CAL frequency changes between IVIG+ASA and IVIG alone groups during study period.

© 2025 Kuo HC et al. *JAMA Network Open*.

eTable 10. Comparison Frequency of Abnormal Coronary Arteries Diameter-Subgroup Populations From Liver, Band, Node, and Young Age Clusters

Subgroup	Group	Abnormal (Z score>2.5)	Baseline	At 6 weeks	At 6 months	P-value[b]
high liver+low band	IVIG alone (N=13)	Yes	3 (23.1)	1 (7.7)	1 (7.7)	0.42
		No	10 (76.9)	12 (92.3)	12 (92.3)	
	IVIG+ASA (N=16)	Yes	3 (18.8)	1 (6.3)	0 (0.0)	
		No	13 (81.2)	15 (93.7)	16 (100.0)	
	P-value[a]		Ref	0.13	0.04	
high liver+high node	IVIG alone (N=13)	Yes	3 (23.1)	1 (7.7)	1 (7.7)	0.42
		No	10 (76.9)	12 (92.3)	12 (92.3)	
	IVIG+ASA (N=16)	Yes	3 (18.8)	1 (6.3)	0 (0.0)	
		No	13 (81.2)	15 (93.7)	16 (100.0)	
	P-value[a]		Ref	0.13	0.04	
high liver+young age	IVIG alone (N=16)	Yes	3 (18.8)	0 (0.0)	1 (6.3)	0.18
		No	13 (81.2)	16 (100.0)	15 (93.7)	
	IVIG+ASA (N=12)	Yes	2 (16.7)	0 (0.0)	0 (0.0)	
		No	10 (83.3)	12 (100.0)	12 (100.0)	
	P-value[a]		Ref	0.02	0.08	
low liver + high band	IVIG alone (N=19)	Yes	0 (0.0)	0 (0.0)	1 (5.3)	>0.99
		No	19 (100.0)	19 (100.0)	18 (94.7)	
	IVIG+ASA (N=12)	Yes	1 (8.3)	0 (0.0)	0 (0.0)	
		No	11 (91.7)	12 (100.0)	12 (100.0)	
	P-value[a]		Ref	0.31	>0.99	
low liver+low band	IVIG alone (N=24)	Yes	3 (12.5)	0 (0.0)	0 (0.0)	0.06
		No	21 (87.5)	24 (100.0)	24 (100.0)	
	IVIG+ASA (N=34)	Yes	5 (14.7)	1 (2.9)	1 (2.9)	
		No	29 (85.3)	33 (97.1)	33 (97.1)	
	P-value[a]		Ref	0.02	0.02	

© 2025 Kuo HC et al. *JAMA Network Open*.

Subgroup	Group	Abnormal (Z score>2.5)	Baseline	At 6 weeks	At 6 months	P-value[b]
low liver+high node	IVIG alone (N=24)	Yes	3 (12.5)	0 (0.0)	0 (0.0)	0.06
		No	21 (87.5)	24 (100.0)	24 (100.0)	
	IVIG+ASA (N=34)	Yes	5 (14.7)	1 (2.9)	1 (2.9)	
		No	29 (85.3)	33 (97.1)	33 (97.1)	
	P-value[a]		Ref	0.02	0.02	
low liver+low node	IVIG alone (N=19)	Yes	0 (0.0)	0 (0.0)	1 (5.3)	>0.99
		No	19 (100.0)	19 (100.0)	18 (94.7)	
	IVIG+ASA (N=12)	Yes	1 (8.3)	0 (0.0)	0 (0.0)	
		No	11 (91.7)	12 (100.0)	12 (100.0)	
	P-value[a]		Ref	0.31	>0.99	
low liver+young age	IVIG alone (N=27)	Yes	2 (7.4)	0 (0.0)	0 (0.0)	0.06
		No	25 (92.6)	27 (100.0)	27 (100.0)	
	IVIG+ASA (N=31)	Yes	4 (12.9)	1 (3.2)	1 (3.2)	
		No	27 (87.1)	30 (96.8)	30 (96.8)	
	P-value[a]		Ref	0.05	0.05	
low liver+old age	IVIG alone (N=16)	Yes	1 (6.3)	0 (0.0)	1 (6.3)	>0.99
		No	15 (93.8)	16 (100.0)	15 (93.8)	
	IVIG+ASA (N=15)	Yes	2 (13.3)	0 (0.0)	0 (0.0)	
		No	13 (86.7)	15 (100.0)	15 (100.0)	
	P-value[a]		Ref	0.08	0.30	
high band+low node	IVIG alone (N=27)	Yes	1 (3.7)	0 (0.0)	1 (3.7)	0.42
		No	26 (96.3)	27 (100.0)	26 (96.3)	
	IVIG+ASA (N=19)	Yes	1 (5.3)	0 (0.0)	0 (0.0)	
		No	18 (94.7)	19 (100.0)	19 (100.0)	
	P-value[a]		Ref	0.15	0.56	

© 2025 Kuo HC et al. *JAMA Network Open*.

Subgroup	Group	Abnormal (Z score>2.5)	Baseline	At 6 weeks	At 6 months	P-value[b]
high band+young age	IVIG alone (N=21)	Yes	1 (4.8)	0 (0.0)	0 (0.0)	>0.99
		No	20 (95.2)	21 (100.0)	21 (100.0)	
	IVIG+ASA (N=15)	Yes	1 (6.7)	0 (0.0)	0 (0.0)	
		No	14 (93.3)	15 (100.0)	15 (100.0)	
	P-value[a]		Ref	0.15	0.15	
low band+high node	IVIG alone (N=38)	Yes	6 (15.8)	1 (2.6)	0 (0.0)	0.06
		No	32 (84.2)	37 (97.4)	38 (100.0)	
	IVIG+ASA (N=50)	Yes	8 (16.0)	2 (4.0)	1 (2.0)	
		No	42 (84.0)	48 (96.0)	49 (98.0)	
	P-value[a]		Ref	0.005	<0.001	
low band+old age	IVIG alone (N=15)	Yes	2 (13.3)	1 (6.7)	0 (0.0)	0.42
		No	13 (86.7)	14 (93.3)	15 (100.0)	
	IVIG+ASA (N=22)	Yes	3 (13.6)	1 (4.5)	0 (0.0)	
		No	19 (86.4)	21 (95.5)	22 (100.0)	
	P-value[a]		Ref	0.23	0.02	
low band+young age	IVIG alone (N=23)	Yes	4 (17.4)	0 (0.0)	1 (4.3)	0.18
		No	19 (82.6)	23 (100.0)	22 (95.7)	
	IVIG+ASA (N=28)	Yes	5 (17.9)	1 (3.6)	1 (3.6)	
		No	23 (82.1)	27 (96.4)	27 (96.4)	
	P-value[a]		Ref	0.008	0.03	
high node+old age	IVIG alone (N=15)	Yes	2 (13.3)	1 (6.7)	0 (0.0)	0.42
		No	13 (86.7)	14 (93.3)	15 (100.0)	
	IVIG+ASA (N=22)	Yes	3 (13.6)	1 (4.5)	0 (0.0)	
		No	19 (86.4)	21 (95.5)	22 (100.0)	
	P-value[a]		Ref	0.23	0.02	

© 2025 Kuo HC et al. *JAMA Network Open*.

Subgroup	Group	Abnormal (Z score>2.5)	Baseline	At 6 weeks	At 6 months	P-value[b]
high node+young age	IVIG alone (N=23)	Yes	4 (17.4)	0 (0.0)	1 (4.3)	0.18
		No	19 (82.6)	23 (100.0)	22 (95.7)	
	IVIG+ASA (N=28)	Yes	5 (17.9)	1 (3.6)	1 (3.6)	
		No	23 (82.1)	27 (96.4)	27 (96.4)	
	P-value[a]		Ref	0.008	0.03	
low node+young age	IVIG alone (N=21)	Yes	1 (4.8)	0 (0.0)	0 (0.0)	>0.99
		No	20 (95.2)	21 (100.0)	21 (100.0)	
	IVIG+ASA (N=15)	Yes	1 (6.7)	0 (0.0)	0 (0.0)	
		No	14 (93.3)	15 (100.0)	15 (100.0)	
	P-value[a]		Ref	0.15	0.15	

[a]CAL frequency difference between the 6th week or the 6th month and baseline; [b]CAL frequency changes between IVIG+ASA and IVIG alone groups during study period.

© 2025 Kuo HC et al. *JAMA Network Open*.

eTable 11. Comparison Frequency of Abnormal Coronary Arteries Diameter-Low CRP Subgroup (CRP ≤ 100 mg/L)

CAL	Group	Abnormal (Z score>2.5)	Baseline	At 6 weeks	At 6 months	P-value[b]
LMCA	IVIG alone	Yes	3 (5.5)	0 (0.0)	0 (0.0)	0.18
		No	52 (94.5)	55 (100.0)	55 (100.0)	
	IVIG+ASA	Yes	4 (8.0)	1 (2.0)	0 (0.0)	
		No	46 (92.0)	49 (98.0)	50 (100.0)	
	P-value[a]		Ref	0.03	0.007	
LAD	IVIG alone	Yes	0 (0.0)	0 (0.0)	0 (0.0)	N.A.
		No	55 (100.0)	55 (100.0)	55 (100.0)	
	IVIG+ASA	Yes	0 (0.0)	0 (0.0)	0 (0.0)	
		No	50 (100.0)	50 (100.0)	50 (100.0)	
	P-value[a]		Ref	N.A.	N.A.	
RCA	IVIG alone	Yes	4 (7.3)	1 (1.8)	1 (1.8)	0.23
		No	51 (92.7)	54 (98.2)	54 (98.2)	
	IVIG+ASA	Yes	2 (4.0)	0 (0.0)	1 (2.0)	
		No	48 (96.0)	50 (100.0)	49 (98.0)	
	P-value[a]		Ref	0.06	0.15	
Overall	IVIG alone	Yes	6 (10.9)	1 (1.8)	1 (1.8)	>0.99
		No	49 (89.1)	54 (98.2)	54 (98.2)	
	IVIG+ASA	Yes	6 (12.0)	1 (2.0)	1 (2.0)	
		No	44 (88.0)	49 (98.0)	49 (98.0)	
	P-value[a]		Ref	0.006	0.006	

LMCA: Left main coronary artery, RCA: Right coronary artery, LAD: Left anterior descending; [a]CAL frequency difference between the 6th week or the 6th month and baseline; [b]CAL frequency changes between IVIG+ASA and IVIG alone groups during study period.

© 2025 Kuo HC et al. *JAMA Network Open*.

eTable 12. Comparison Frequency of Abnormal Coronary Arteries Diameter-High CRP Subgroup (CRP > 100 mg/L)

CAL	Group	Abnormal (Z score>2.5)	Baseline	At 6 weeks	At 6 months	P-value[b]
LMCA	IVIG alone	Yes	1 (1.0)	0 (0.0)	1 (1.0)	0.67
		No	9 (90.0)	10 (100.0)	9 (90.0)	
	IVIG+ASA	Yes	2 (10.5)	1 (5.3)	0 (0.0)	
		No	17 (89.5)	18 (94.7)	19 (100.0)	
	P-value[a]		Ref	0.30	0.30	
LAD	IVIG alone	Yes	0 (0.0)	0 (0.0)	0 (0.0)	N.A.
		No	10 (100.0)	10 (100.0)	10 (100.0)	
	IVIG+ASA	Yes	0 (0.0)	0 (0.0)	0 (0.0)	
		No	19 (100.0)	19 (100.0)	19 (100.0)	
	P-value[a]		Ref	N.A.	N.A.	
RCA	IVIG alone	Yes	0 (0.0)	0 (0.0)	0 (0.0)	0.18
		No	10 (100.0)	10 (100.0)	10 (100.0)	
	IVIG+ASA	Yes	1 (5.3)	1 (5.3)	0 (0.0)	
		No	18 (94.7)	18 (94.7)	19 (100.0)	
	P-value[a]		Ref	>0.99	0.31	
Overall	IVIG alone	Yes	1 (1.0)	0 (0.0)	1 (1.0)	0.53
		No	9 (90.0)	10 (100.0)	9 (90.0)	
	IVIG+ASA	Yes	3 (15.8)	1 (5.3)	0 (0.0)	
		No	16 (84.2)	18 (94.7)	19 (100.0)	
	P-value[a]		Ref	0.16	0.16	

LMCA: Left main coronary artery, RCA: Right coronary artery, LAD: Left anterior descending; [a]CAL frequency difference between the 6th week or the 6th month and baseline; [b]CAL frequency changes between IVIG+ASA and IVIG alone groups during study period.

© 2025 Kuo HC et al. *JAMA Network Open*.

eTable 13. Comparison Frequency of Abnormal Coronary Arteries Diameter-Low Platelet Count Subgroup (Platelet < 400 × 10^3/µL)

CAL	Group	Abnormal (Z score>2.5)	Baseline	At 6 weeks	At 6 months	P-value[b]
LMCA	IVIG alone	Yes	4 (7.4)	0 (0.0)	1 (1.9)	0.53
		No	50 (92.6)	54 (100.0)	53 (98.1)	
	IVIG+ASA	Yes	5 (10.9)	2 (4.3)	0 (0.0)	
		No	41 (89.1)	44 (95.7)	46 (100.0)	
	P-value[a]		Ref	0.03	0.009	
LAD	IVIG alone	Yes	0 (0.0)	0 (0.0)	0 (0.0)	N.A.
		No	54 (100.0)	54 (100.0)	54 (100.0)	
	IVIG+ASA	Yes	0 (0.0)	0 (0.0)	0 (0.0)	
		No	46 (100.0)	46 (100.0)	46 (100.0)	
	P-value[a]		Ref	N.A.	N.A.	
RCA	IVIG alone	Yes	3 (5.6)	1 (1.9)	1 (1.9)	0.42
		No	51 (94.4)	53 (98.1)	53 (98.1)	
	IVIG+ASA	Yes	3 (6.5)	1 (2.2)	0 (0.0)	
		No	43 (93.5)	45 (97.8)	46 (100.0)	
	P-value[a]		Ref	0.15	0.05	
Overall	IVIG alone	Yes	6 (11.1)	1 (1.9)	2 (3.7)	0.81
		No	48 (88.9)	53 (98.1)	52 (96.3)	
	IVIG+ASA	Yes	8 (17.4)	2 (4.3)	0 (0.0)	
		No	38 (82.6)	44 (95.7)	46 (100.0)	
	P-value[a]		Ref	0.005	0.002	

LMCA: Left main coronary artery, RCA: Right coronary artery, LAD: Left anterior descending; [a]CAL frequency difference between the 6th week or the 6th month and baseline; [b]CAL frequency changes between IVIG+ASA and IVIG alone groups during study period.

© 2025 Kuo HC et al. *JAMA Network Open*.

eTable 14. Comparison Frequency of Abnormal Coronary Arteries Diameter-High Platelet Count Subgroup (Platelet ≥ 400 × 10^3/μL)

CAL	Group	Abnormal (Z score>2.5)	Baseline	At 6 weeks	At 6 months	P-value[b]
LMCA	IVIG alone	Yes	0 (0.0)	0 (0.0)	0 (0.0)	0.42
		No	11 (100.0)	11 (100.0)	11 (100.0)	
	IVIG+ASA	Yes	1 (4.3)	0 (0.0)	0 (0.0)	
		No	22 (95.7)	23 (100.0)	23 (100.0)	
	P-value[a]		Ref	0.31	0.31	
LAD	IVIG alone	Yes	0 (0.0)	0 (0.0)	0 (0.0)	N.A.
		No	11 (100.0)	11 (100.0)	11 (100.0)	
	IVIG+ASA	Yes	0 (0.0)	0 (0.0)	0 (0.0)	
		No	23 (100.0)	23 (100.0)	23 (100.0)	
	P-value[a]		Ref	N.A.	N.A.	
RCA	IVIG alone	Yes	1 (9.1)	0 (0.0)	0 (0.0)	>0.99
		No	10 (90.9)	11 (100.0)	11 (100.0)	
	IVIG+ASA	Yes	0 (0.0)	0 (0.0)	1 (4.3)	
		No	23 (100.0)	23 (100.0)	22 (95.7)	
	P-value[a]		Ref	0.31	0.31	
Overall	IVIG alone	Yes	1 (9.1)	0 (0.0)	0 (0.0)	0.42
		No	10 (90.9)	11 (100.0)	11 (100.0)	
	IVIG+ASA	Yes	1 (4.3)	0 (0.0)	1 (4.3)	
		No	22 (95.7)	23 (100.0)	22 (95.7)	
	P-value[a]		Ref	0.15	0.56	

LMCA: Left main coronary artery, RCA: Right coronary artery, LAD: Left anterior descending; [a]CAL frequency difference between the 6th week or the 6th months and baseline; [b]CAL frequency changes between IVIG+ASA and IVIG alone groups during study period.

© 2025 Kuo HC et al. *JAMA Network Open*.

eReferences

1. Akagi T, Rose V, Benson LN, Newman A, Freedom RM. Outcome of coronary artery aneurysms after Kawasaki disease. J Pediatr. 1992;121(5 Pt 1):689-94. doi: 10.1016/s0022-3476(05)81894-3. PubMed PMID: 1432415.
2. Shulman ST, De Inocencio J, Hirsch R. Kawasaki disease. Pediatr Clin North Am. 1995;42(5):1205-22. doi: 10.1016/s0031-3955(16)40059-3. PubMed PMID: 7567192.
3. Yu HR, Kuo HC, Sheen JM, Wang L, Lin IC, Wang CL, Yang KD. A unique plasma proteomic profiling with imbalanced fibrinogen cascade in patients with Kawasaki disease. Pediatr Allergy Immunol. 2009;20(7):699-707. Epub 20090112. doi: 10.1111/j.1399-3038.2008.00844.x. PubMed PMID: 19170925.
4. Wu MT, Hsieh KS, Lin CC, Yang CF, Pan HB. Images in cardiovascular medicine. Evaluation of coronary artery aneurysms in Kawasaki disease by multislice computed tomographic coronary angiography. Circulation. 2004;110(14):e339. doi: 10.1161/01.CIR.0000143374.80173.EF. PubMed PMID: 15466653.
5. Weng KP, Ho TY, Chiao YH, Cheng JT, Hsieh KS, Huang SH, Ou SF, Liu KH, Hsu CJ, Lu PJ, Hsiao M, Ger LP. Cytokine genetic polymorphisms and susceptibility to Kawasaki disease in Taiwanese children. Circ J. 2010;74(12):2726-33. Epub 20101030. doi: 10.1253/circj.cj-10-0542. PubMed PMID: 21048327.
6. Weng KP, Hsieh KS, Ho TY, Huang SH, Lai CR, Chiu YT, Huang SC, Lin CC, Hwang YT, Ger LP. IL-1B polymorphism in association with initial intravenous immunoglobulin treatment failure in Taiwanese children with Kawasaki disease. Circ J. 2010;74(3):544-51. Epub 20100118. doi: 10.1253/circj.cj-09-0664. PubMed PMID: 20081319.
7. Weng KP, Hsieh KS, Hwang YT, Huang SH, Lai TJ, Yuh YS, Hou YY, Lin CC, Huang SC, Chang CK, Lin MW, Ger LP. IL-10 polymorphisms are associated with coronary artery lesions in acute stage of Kawasaki disease. Circ J. 2010;74(5):983-9. Epub 20100326. doi: 10.1253/circj.cj-09-0801. PubMed PMID: 20339193.
8. Kuo HC, Guo MM, Lo MH, Hsieh KS, Huang YH. Effectiveness of intravenous immunoglobulin alone and intravenous immunoglobulin combined with high-dose aspirin in the acute stage of Kawasaki disease: study protocol for a randomized controlled trial. BMC Pediatr. 2018;18(1):200. Epub 20180622. doi: 10.1186/s12887-018-1180-1. PubMed PMID: 29933749; PMCID: PMC6015467.

© 2025 Kuo HC et al. *JAMA Network Open*.
