# Supplementary material for: Intravenous Immunoglobulin Alone for Coronary Artery Lesion Treatment of Kawasaki Disease: A Randomized Clinical Trial
Source: JAMA Netw Open. 2025 Apr 3;8(4):e253063. doi: 10.1001/jamanetworkopen.2025.3063 (PMC11969286; doi:10.1001/jamanetworkopen.2025.3063)
Supplement: Supplement 3. — Data Sharing Statement [file jamanetwopen-e253063-s003.pdf]

# Data Sharing Statement

Kuo. Intravenous Immunoglobulin Alone for Coronary Artery Lesion Treatment of Kawasaki Disease. *JAMA Netw Open*. Published April 03, 2025.
doi:10.1001/jamanetworkopen.2025.3063

**Data**
**Additional Information:** clinicaltrials.gov identifier: NCT02951234
**Data available:** No
